# Supplementary material for: A salt-free medium facilitating electrode prelithiation towards fast-charging and high-energy lithium-ion batteries
Source: Nat Commun. 2025 Aug 26;16:7956. doi: 10.1038/s41467-025-63257-w (PMC12381243; doi:10.1038/s41467-025-63257-w)
Supplement: Supplementary file 1 — Supplementary information [file 41467_2025_63257_MOESM1_ESM.pdf]

# A salt-free medium facilitating electrode prelithiation towards fast-charging and high-energy lithium-ion batteries

Yangtao Ou, Bao Zhang, Renming Zhan, Shiyu Liu, Wenyu Wang, Shuibin Tu, Yang Hu, Zihe Chen, Xiangrui Duan, Xiancheng Wang, Li Wang & Yongming Sun*

Corresponding author: Yongming Sun, yongmingsun@hust.edu.cn

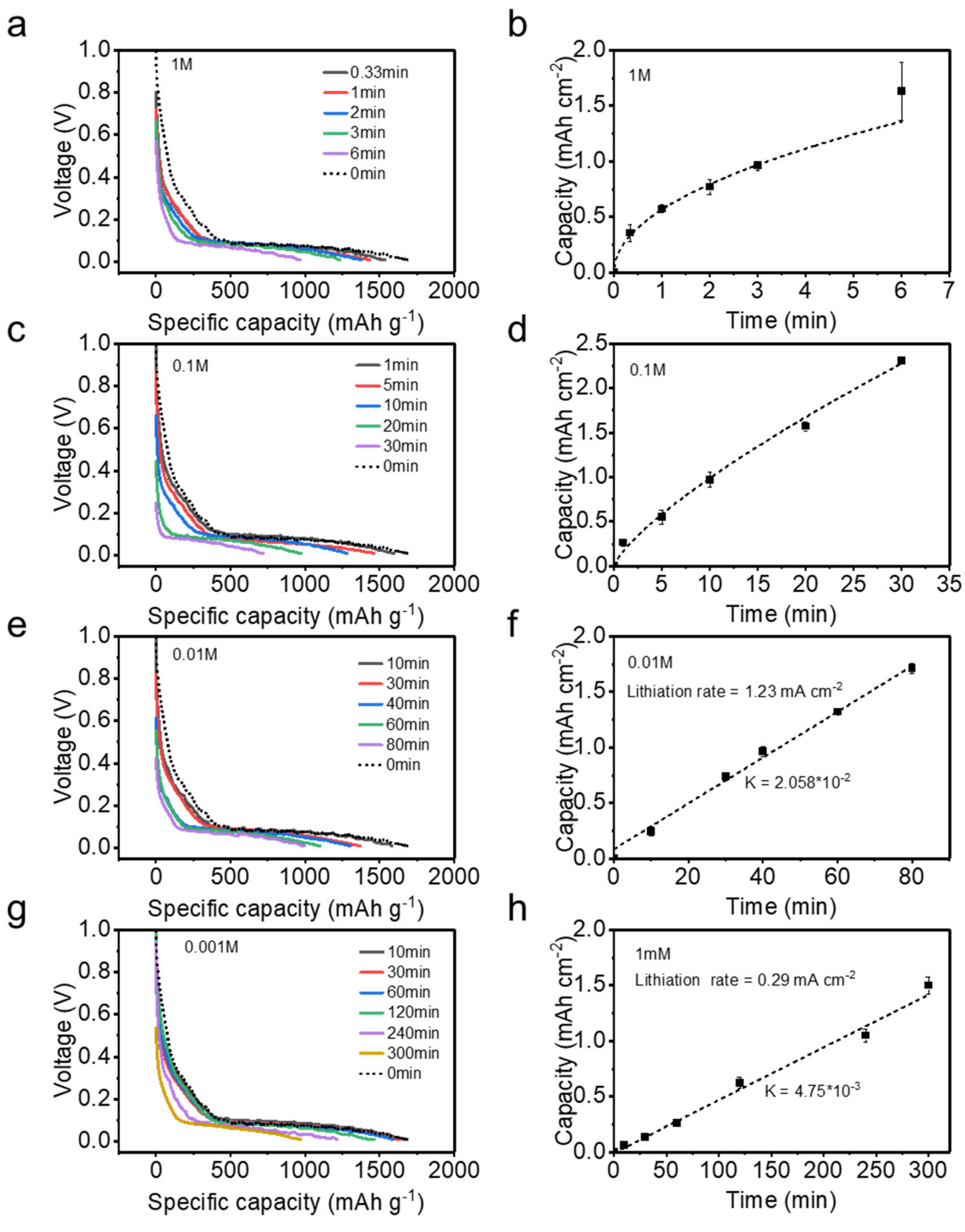


**Supplementary Fig. 1 | Contact prelithiation of Si/C electrodes using electrolytes with various concentrations. a-h**, The first-cycle discharge voltage profiles and the corresponding prelithiation capacity-time plots of the Si/C electrode after contact prelithiation using 1 M (**a**, **b**), 0.1 M (**c**, **d**), 0.01 M (**e**, **f**) and 0.001 M (**g**, **h**) $LiPF_6$ electrolytes. After the prelithiation operation, all the Si/C electrodes were further electrochemically lithiated to 0.01 V (*vs.* $Li^+/Li$) at 0.1 *C*. The difference between the

lithiation capacity of a fresh Si/C electrode and the electrochemical lithiation capacity of the prelithiated Si/C electrode was used to evaluate prelithiation capacity.

To verify the above analysis, we explored the Li metal-contact prelithiation of Si/C electrodes in electrolyte mediums with different $Li^+$ concentrations (1 M, 0.1 M, 0.01 M and 0.001 M $LiPF_6$). As shown in Supplementary Fig. 1 and Supplementary Table 1, the Si/C electrode demonstrated rapid prelithiation and large inhomogeneity over the reaction time using 1 M $LiPF_6$ medium, but it showed a marked deceleration in reaction rate and improvement in reaction homogeneity over the reaction time in response to the decrease in electrolyte concentration. Interestingly, the prelithiation capacity showed entirely linear dependence over the reaction time when the $Li^+$ concentration dropped to 0.01 M. As shown in Supplementary Figs. 1a, b, the Si/C electrode delivered average prelithiation capacities of 0.35, 0.57, 0.77, 0.96 and 1.63 mAh $cm^{-2}$ at 0.33, 1, 2, 3 and 6 min using 1 M $LiPF_6$, thereby demonstrating rapid lithiation reaction kinetics. It is observed that the increment of prelithiation capacity exhibited a gradual decrease as the reaction progressed, thereby demonstrating temporal non-uniformity. It is noteworthy that the value of prelithiation capacity in different electrolyte mediums after same reaction time showed a marked deceleration in response to the decrease in electrolyte concentration. As the $Li^+$ concentration was reduced to 0.1 M, the prelithiation capacity progressively transitioned to a linear correlation with the prelithiation time (Supplementary Figs. 1c, d). This response became entirely linear when the $Li^+$ concentration dropped to 0.01 M (Supplementary Figs. 1e, f). Typically, the prelithiation rates in 0.01 M and 0.001 M $LiPF_6$ were calculated as 1.23 mA $cm^{-2}$ and 0.29 mA $cm^{-2}$, respectively (Supplementary Figs. 1g, h). These results indicate that a lower $Li^+$ concentration in the electrolyte medium would invariably reduce the prelithiation reaction rate and enhance the temporal uniformity of contact prelithiation.

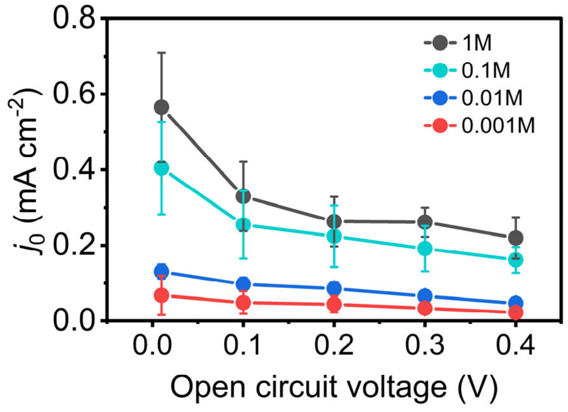


**Supplementary Fig. 2 | The measured exchange current density using electrolytes with various $Li^+$ concentrations at various open circuit voltage (OCV) values (from 0.4 V to 0.01 V) during discharging.**

The exchange current density ($j_0$) was further characterized to analyze the electrochemical reaction kinetics and the charge-transfer reaction rate in electrolytes with different $Li^+$ concentrations at various open circuit voltages[1] (OCVs, Supplementary Fig. 2). As the OCV decreased from 0.4 to 0.01 V, the $j_0$ values for 1M $LiPF_6$ increased from ~0.22 to ~0.57 mA cm$^{-2}$, a factor of 2.6. The large $j_0$ values and their significant change with the OCV contribute to the uncontrollability of the prelithiation reaction for practical application. The overall $j_0$ values rapidly decreased with the reduction of the electrolyte concentration from 1 to 0.01 M and showed only a slight change from 0.01 to 0.001 M, demonstrating their differences in interfacial charge-transfer kinetics for electrolyte with different $Li^+$ concentrations. Notably, the $j_0$ value at 0.01 V of the 0.001 M $LiPF_6$ electrolyte was only 1/8.4 that of the 1 M $LiPF_6$ electrolyte. It should be emphasized that the Si/C electrodes in low $Li^+$ ion-concentration electrolytes exhibited good stability in $j_0$ values at different OCVs. For instance, the Si/C electrodes in 0.001 M $LiPF_6$ exhibited $j_0$ values of 0.022, 0.034, 0.044, 0.048, and 0.068 mA cm$^{-2}$, providing the smallest difference/fluctuation over the entire prelithiation process. Therefore, ultralow $Li^+$ concentrations of electrolytes (< 0.01 M) can effectively decrease electrochemical reaction kinetics to enable uniform spontaneous prelithiation across the electrode, avoiding electrode or material damage suffered in present electrolyte-mediated prelithiation (EM-prelithiation).

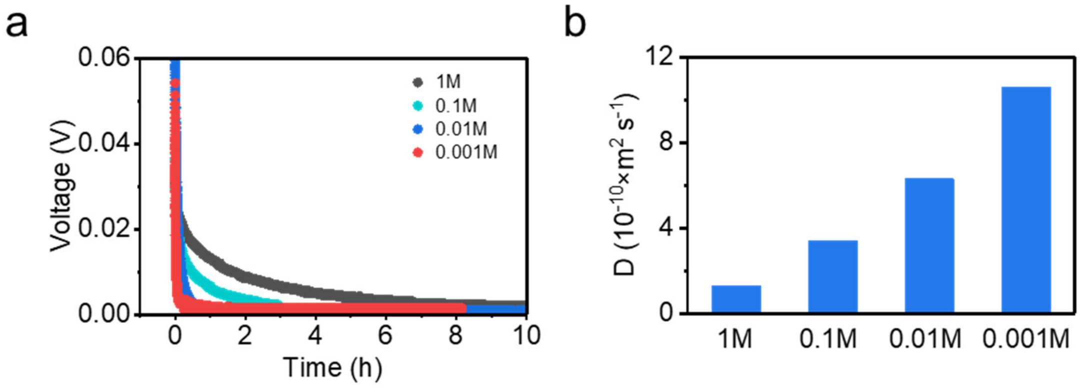


**Supplementary Fig. 3 | Voltage response of a potentiostatic polarization experiment (a) for $LiPF_6$ electrolytes with various concentrations and the corresponding calculated D values (b).**

To characterize the $Li^+$ diffusion coefficient ($D$) in electrolytes with varying $LiPF_6$ concentrations, a combination of steady-state polarization and relaxation-restricted diffusion was conducted[2] (Supplementary Fig. 3). The results revealed that the $D$ values markedly increased from $1.3\times 10^{-10}$ $m^2$ $s^{-1}$ to $10.6 \times 10^{-10}$ $m^2$ $s^{-1}$ (8.2 times) for 1 M $LiPF_6$ and 0.001 M $LiPF_6$, respectively. Thus, an ultralow Li salt concentration condition possesses a significantly faster ion diffusion capability over the electrode thickness, which promotes the spatial uniformity of the prelithiation reaction.

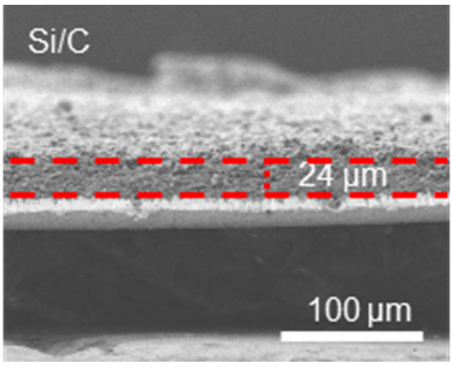


**Supplementary Fig. 4 | Cross-section SEM image of the Si/C electrode.**

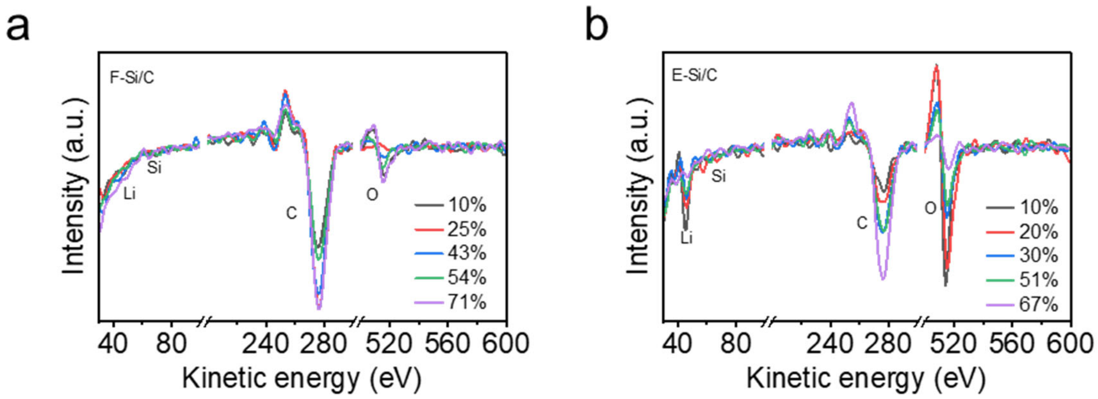


**Supplementary Fig. 5 | AES spectra for the F-Si/C (a) and E-Si/C (b) electrodes at different electrode depths, respectively.**

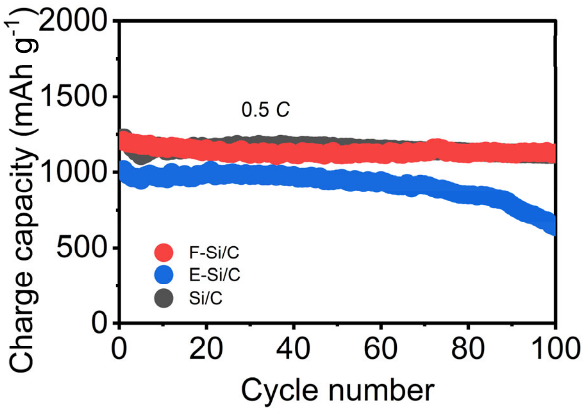


**Supplementary Fig. 6. The cycling performance of the Si/C, F-Si/C and E-Si/C electrodes.**

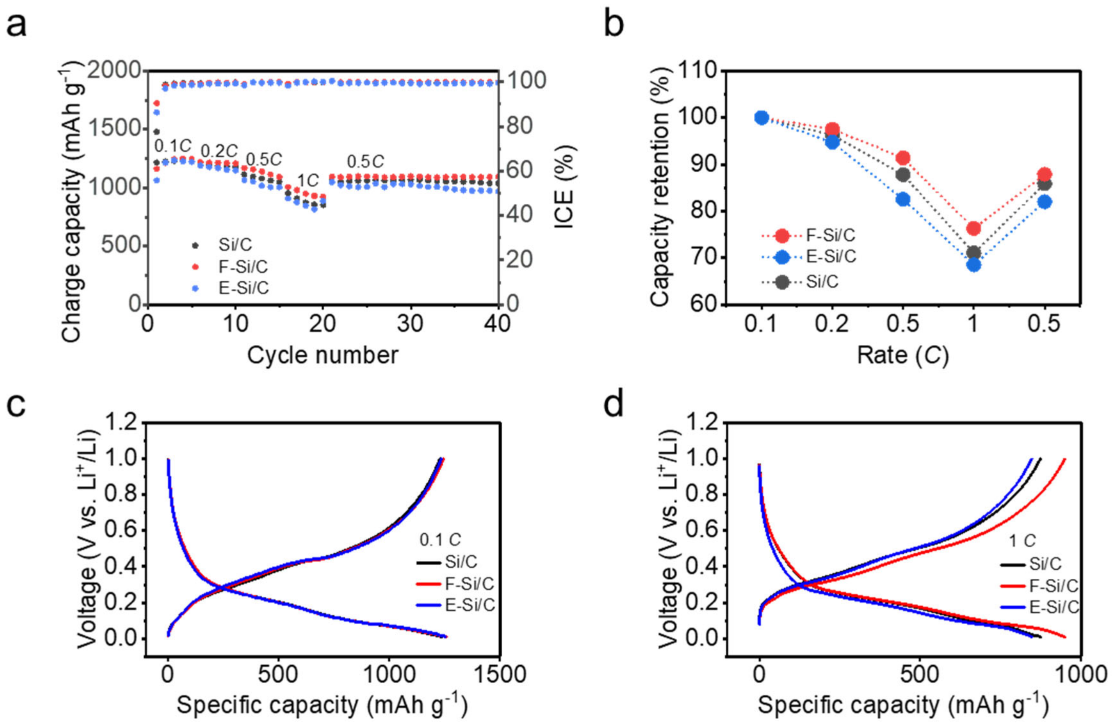


**Supplementary Fig. 7 | The rate performance of the prelithiated Si/C electrodes.** **a**,**b**, The rate performance of the Si/C, F-Si/C and E-Si/C electrodes (**a**), and the corresponding comparison(**b**) **c**,**d**, The voltage profiles of the Si/C, F-Si/C and E-Si/C electrodes at 0.1 *C* (**c**) and 1 *C* (**d**).

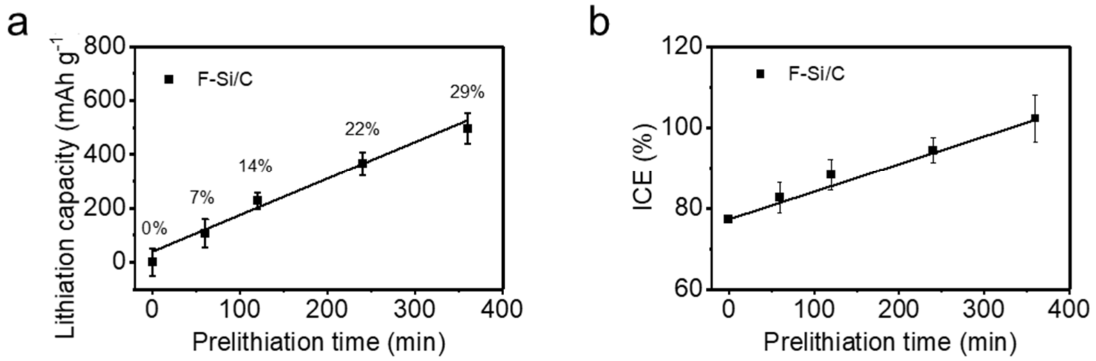


**Supplementary Fig. 8 | Electrochemical performance of the prelithiated Si/C electrodes. a**,**b**, Lithiation capacity values (**a**) and Initial Coulombic efficiency (ICE) (**b**) of the F-Si/C electrodes with different contact prelithiation times. The ratio of the difference of discharge capacity between the fresh Si/C and the prelithiated Si/C and the discharge capacity of fresh Si/C was used to evaluate the prelithiation degree.

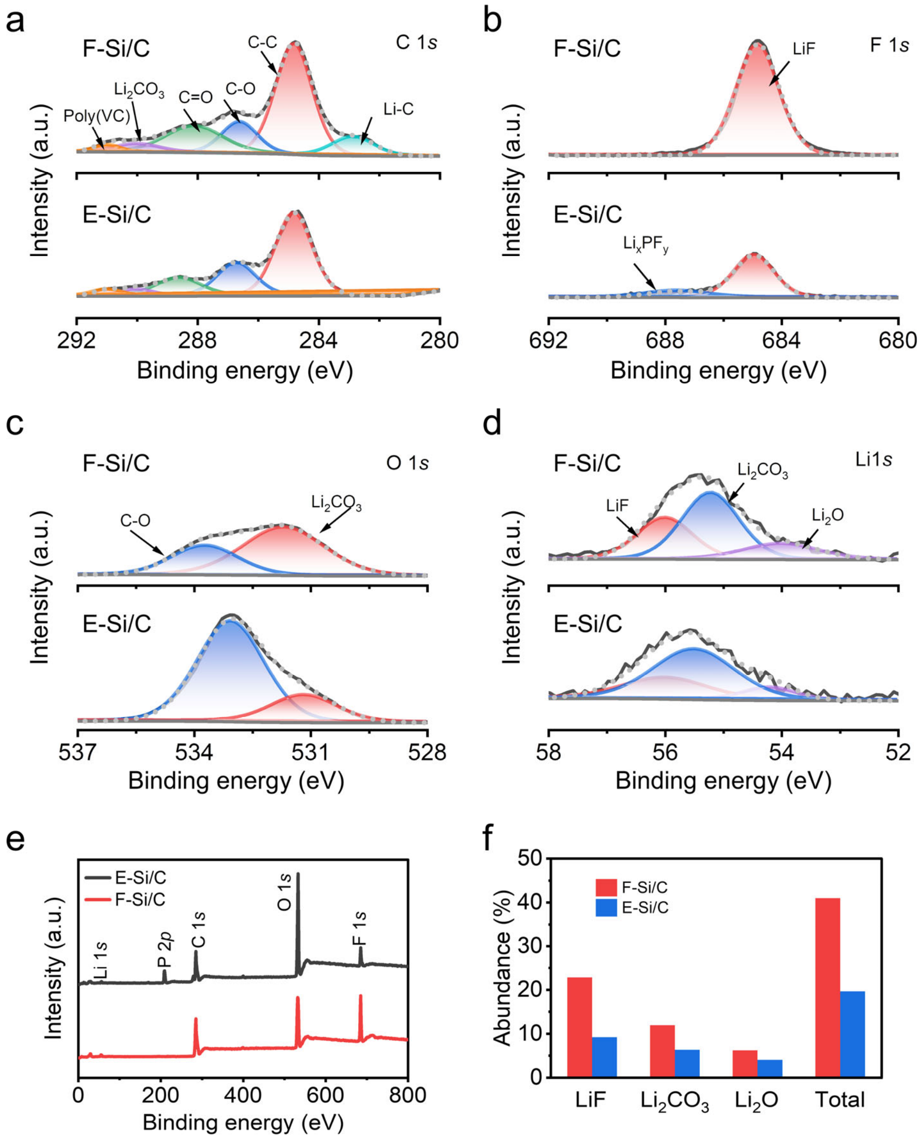


**Supplementary Fig. 9 | XPS spectra of the F-Si/C and E-Si/C electrodes.** a-e, High-resolution C 1*s* (a), F 1*s* (b), O 1*s* (c) and Li 1*s* (d) XPS spectra and survey XPS spectra (e) of the F-Si/C and E-Si/C electrodes. f, The corresponding content analysis of various SEI components.

Based on XPS analysis, a preformed SEI was observed on the F-Si/C electrode, displaying a markedly higher proportion of inorganic species—namely LiF, $Li_2CO_3$, and $Li_2O$—relative to that on the E-Si/C electrode.

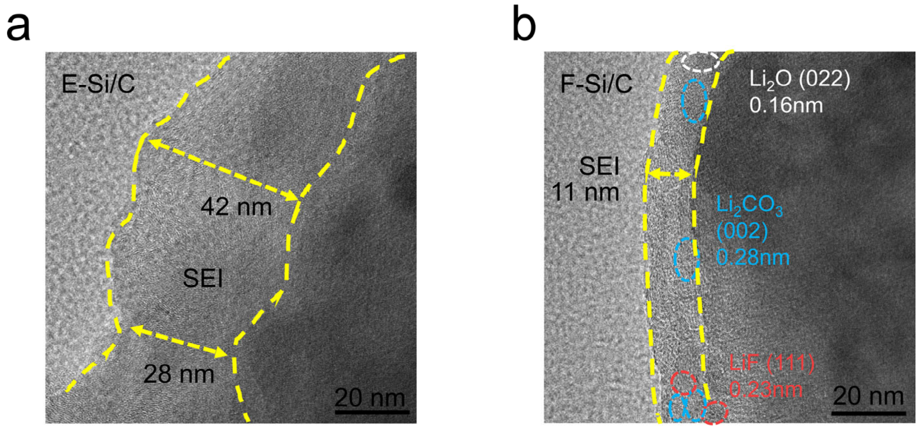


**Supplementary Fig. 10 | High-resolution TEM images for the E-Si/C (a) and F-Si/C (b).** The prelithiation degree of prelithiated Si/C electrodes was ~25%.

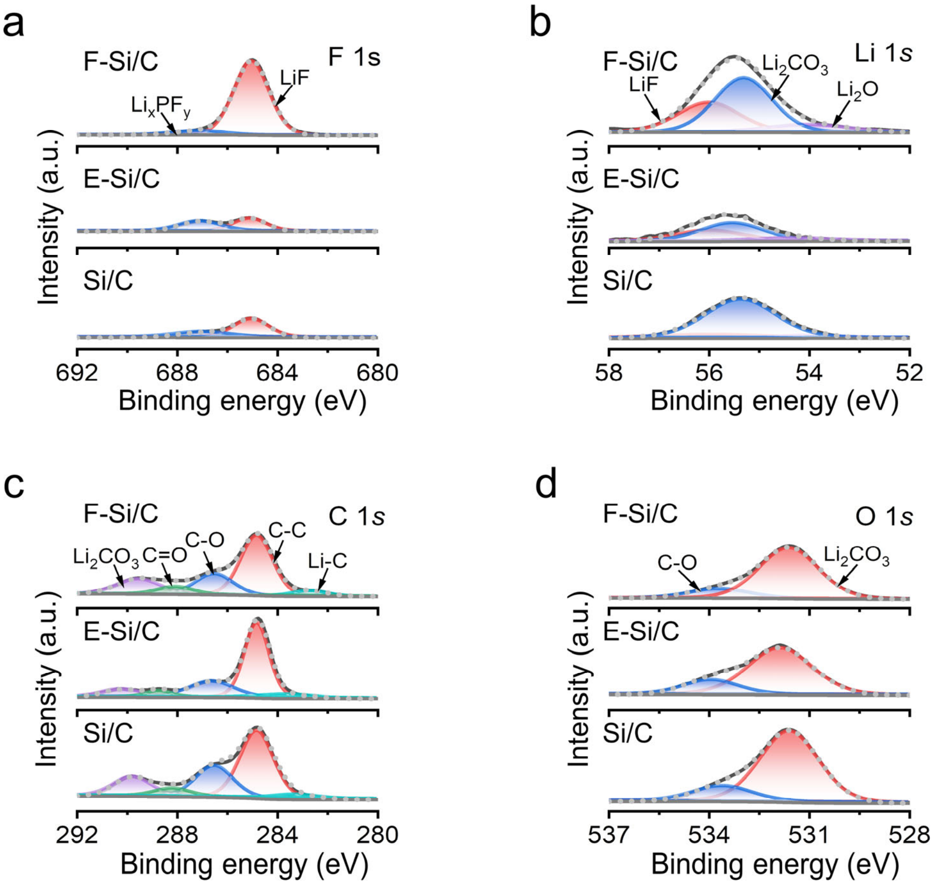


**Supplementary Fig. 11 | High-resolution Li 1*s* (a), F 1*s* (b), C 1*s* (c), and O 1*s* (d) XPS spectra of the F-Si/C, E-Si/C and Si/C electrodes after discharge/charge cycling.**

LiF, $Li_2O$ and $Li_2CO_3$ species constitute the total inorganic components, and their corresponding characteristic peaks are as follows: LiF at 685 eV in the high-resolution F 1s spectrum, $Li_2CO_3$ at 290 eV in the high-resolution C 1s spectrum, and $Li_2O$ at 54 eV in the high-resolution Li 1s spectrum.

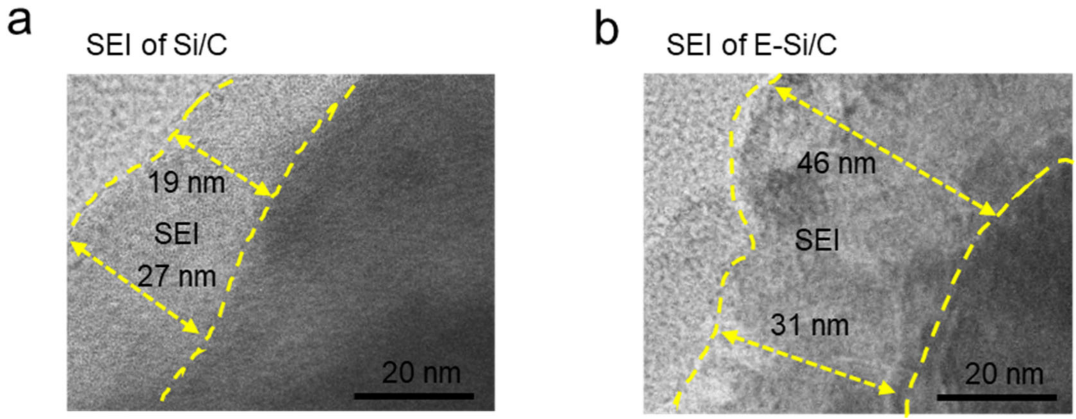


**Supplementary Fig. 12 | High-resolution TEM image of Si/C (a) and E-Si/C (b) after discharge/charge cycling.**

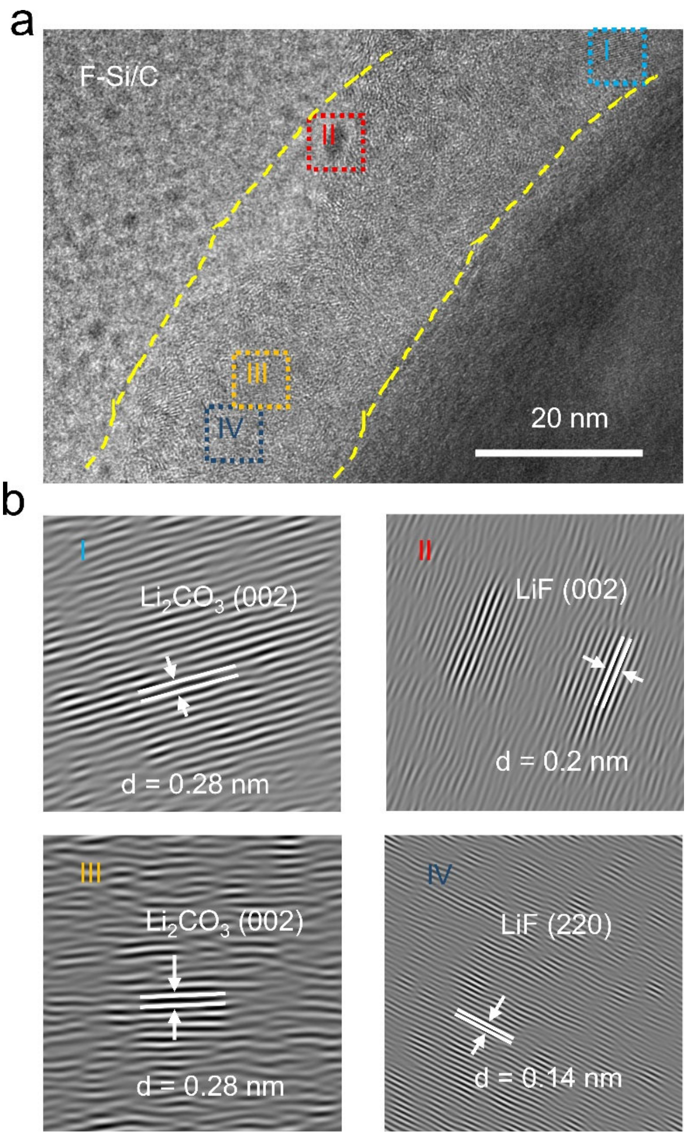


**Supplementary Fig. 13 | HRTEM image for an F-Si/C particle (a) and the corresponding Fourier transform images in the labeled areas (b).**

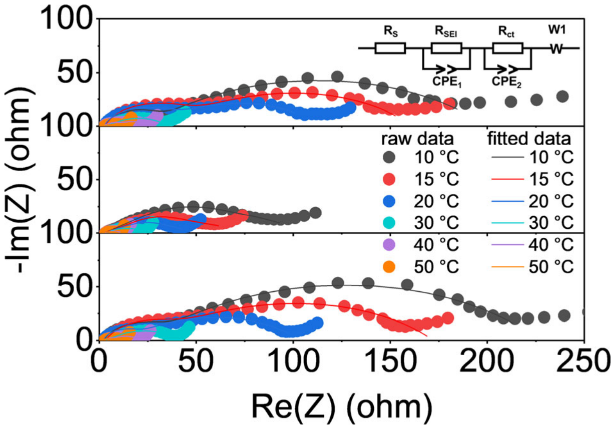


**Supplementary Fig. 14 | EIS spectra of the Si/C, F-Si/C and E-Si/C electrodes tested at different temperatures ranging from 10–50 ºC.**

The semicircular in the low-frequency region corresponds to the charge transfer resistance ($R_{ct}$), and the semicircular in the high-frequency region could be attributed to the SEI resistance ($R_{SEI}$). The activation energies were calculated by the classic Arrhenius law according to the EIS results.

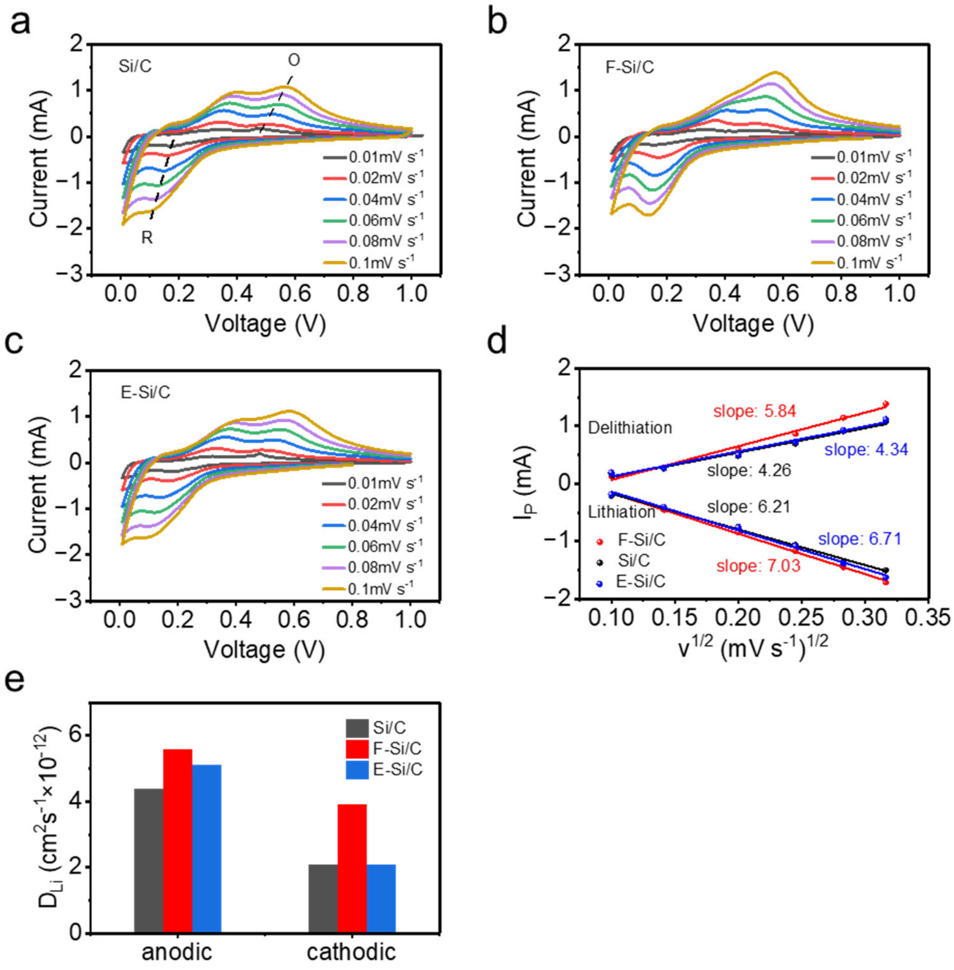


**Supplementary Fig. 15 | The CV curves of the Si/C, F-Si/C and E-Si/C electrodes at different scan rates. a-c,** The CV curves of the Si/C (**a**), F-Si/C (**b**) and E-Si/C (**c**) electrodes at different sweep rates from 0.01 to 0.1 mV $s^{-1}$ in a voltage of 1–0.01 V. **d**, The relationship between the peak current and the square root of the scan rate ($v^{0.5}$) for CV curves of the Si/C, F-Si/C and E-Si/C electrodes at different scan rates. **e**, Comparison of the apparent $Li^+$ diffusion coefficients of the Si/C, F-Si/C and E-Si/C electrodes at anodic/cathodic processes.

The calculated $Li^+$ diffusion coefficients ($D_{Li^+}$) at the anodic/cathodic process of the F-Si/C electrode (5.6/3.9 × $10^{-12}$ $cm^2$ $s^{-1}$) were higher than those of the Si/C electrode (4.4/2.1 × $10^{-12}$ $cm^2$ $s^{-1}$) and the E-Si/C electrode (5.1/2.1 × $10^{-12}$ $cm^2$ $s^{-1}$), suggesting that the F-Si/C electrode with inorganic-rich SEI had faster $Li^+$ diffusion capability.

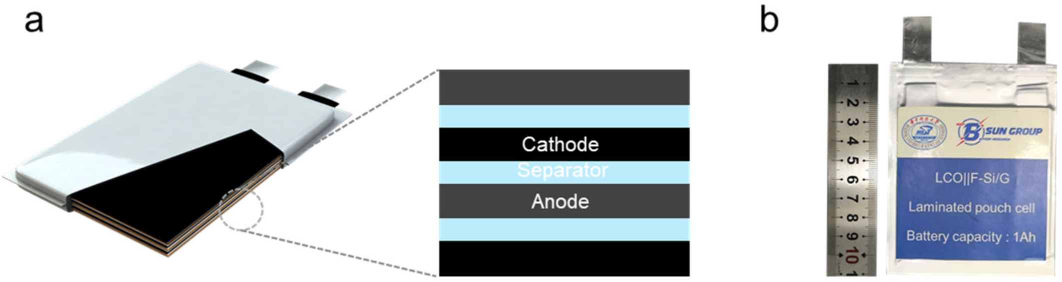


**Supplementary Fig. 16 | Configuration and photo of the pouch cells for electrochemical measurement.**

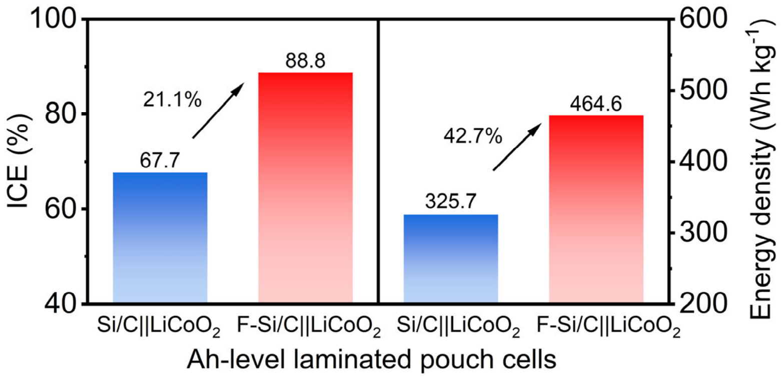


**Supplementary Fig. 17 | Comparison of the ICEs and energy densities of the Si/C||$LiCoO_2$ and Si/C||F-$LiCoO_2$ cells.**

The energy density calculated based on the electrochemical data at 0.1 *C* and the total weight of cathode and anode materials, including active material, binder and conductive additive.

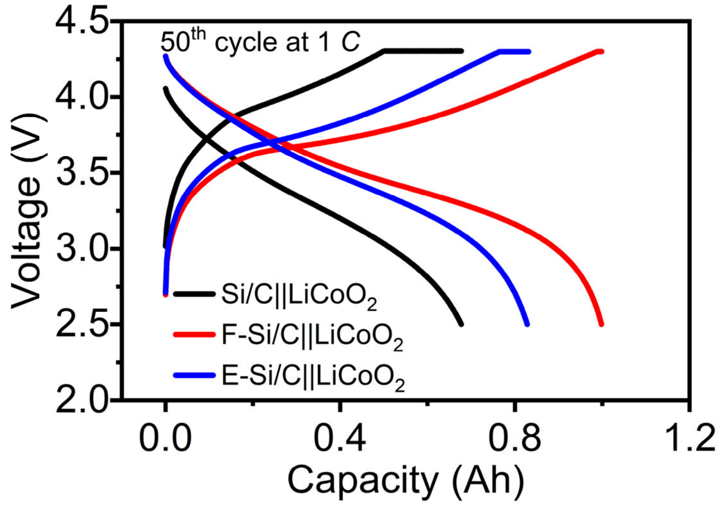


**Supplementary Fig. 18 | The voltage profiles of the Si/C||$LiCoO_2$, Si/C||F-$LiCoO_2$ and Si/C||E-$LiCoO_2$ cells at 1 *C* for the 50$^{th}$ cycle.**

The $LiCoO_2$||F-Si/C cells exhibited the highest average discharge voltage among the tested cells (3.435 V, 3.442 V and 3.408 V for Si/C||$LiCoO_2$, Si/C||F-$LiCoO_2$ and Si/C||E-$LiCoO_2$ cells). Thus, no negative effect on cell polarization was observed for the FEC-mediated prelithiation (FM-prelithiation) of Si/C anode.

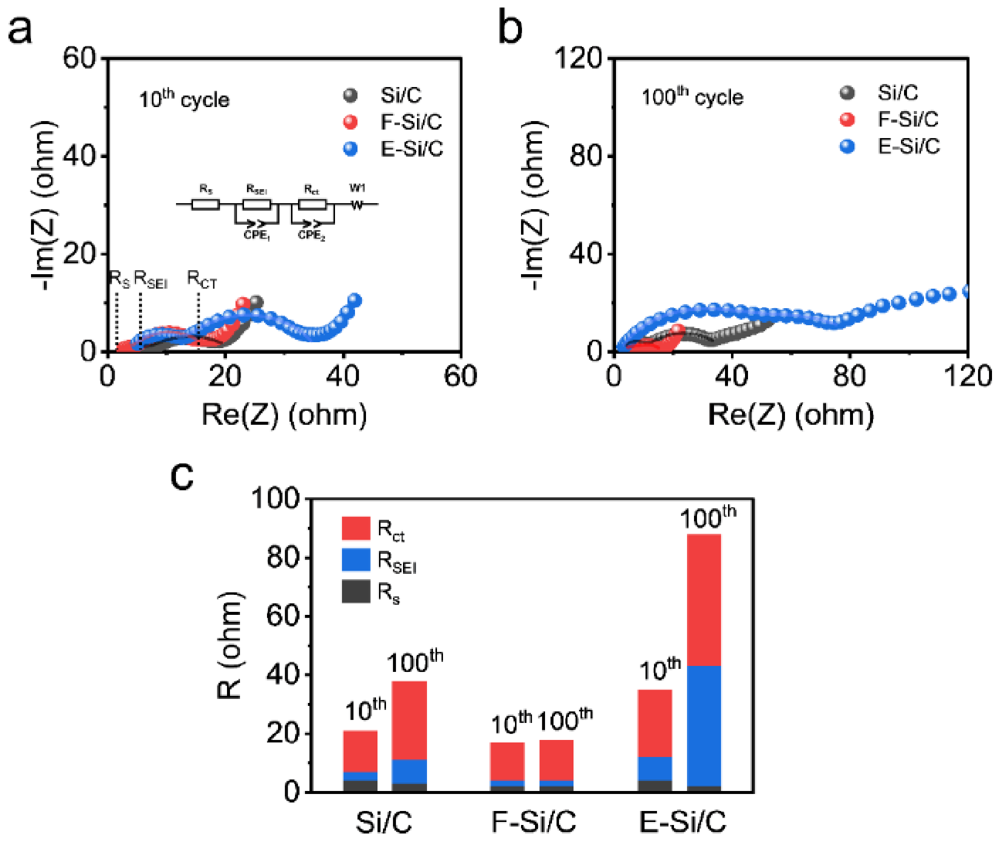


**Supplementary Fig. 19 | EIS spectra of the prelithiated Si/C electrodes. a-b, EIS spectra of the Si/C, F-Si/C and E-Si/C electrodes after the 10$^{th}$ (a) and 100$^{th}$ (b) cycles. c, Comparison of the corresponding impendence values.**

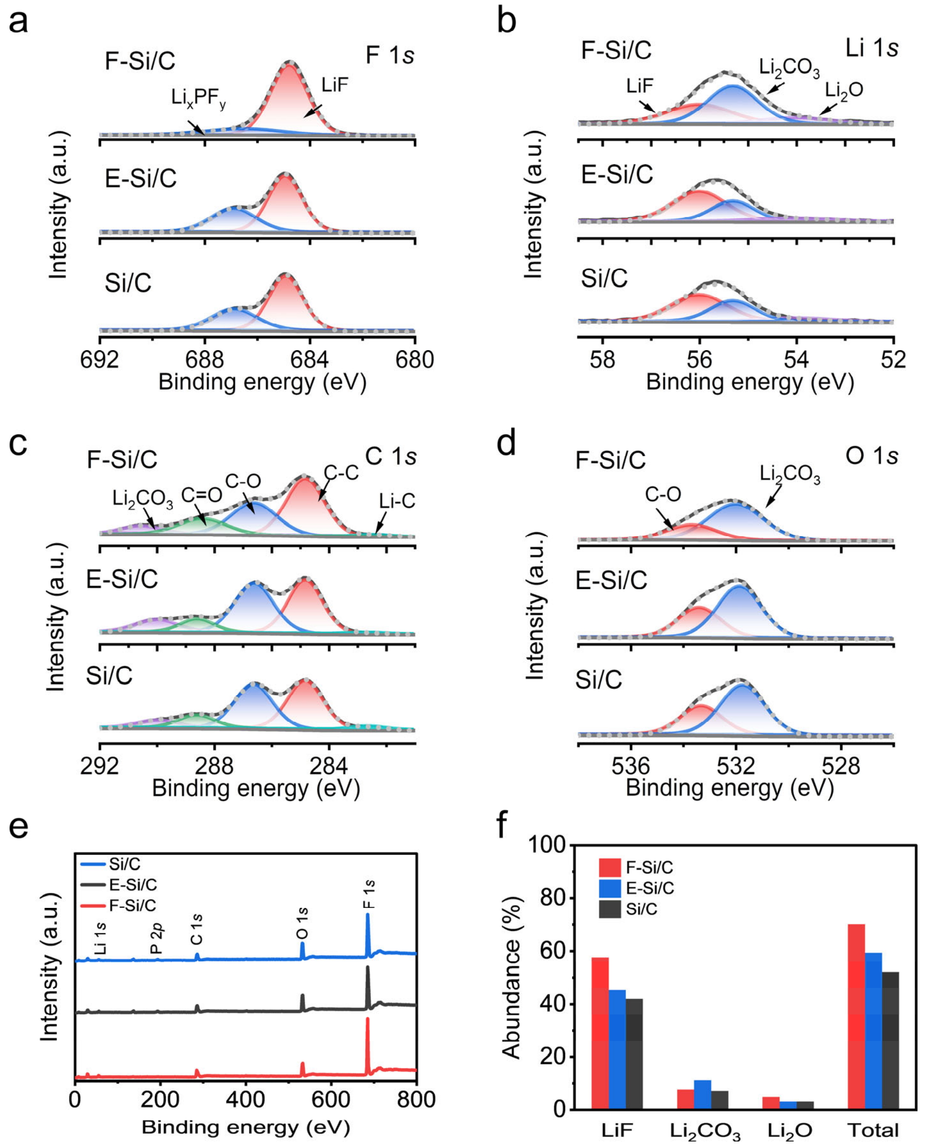


**Supplementary Fig. 20 | XPS spectra of the Si/C, F-Si/C and E-Si/C electrodes after 100 cycles. a-e, High-resolution C 1*s* (a), F 1*s* (b), O 1*s* (c) and Li 1*s* (d) XPS spectra and survey XPS spectra (e) of the Si/C, F-Si/C and E-Si/C electrodes. f, The corresponding content analysis of various SEI components.**

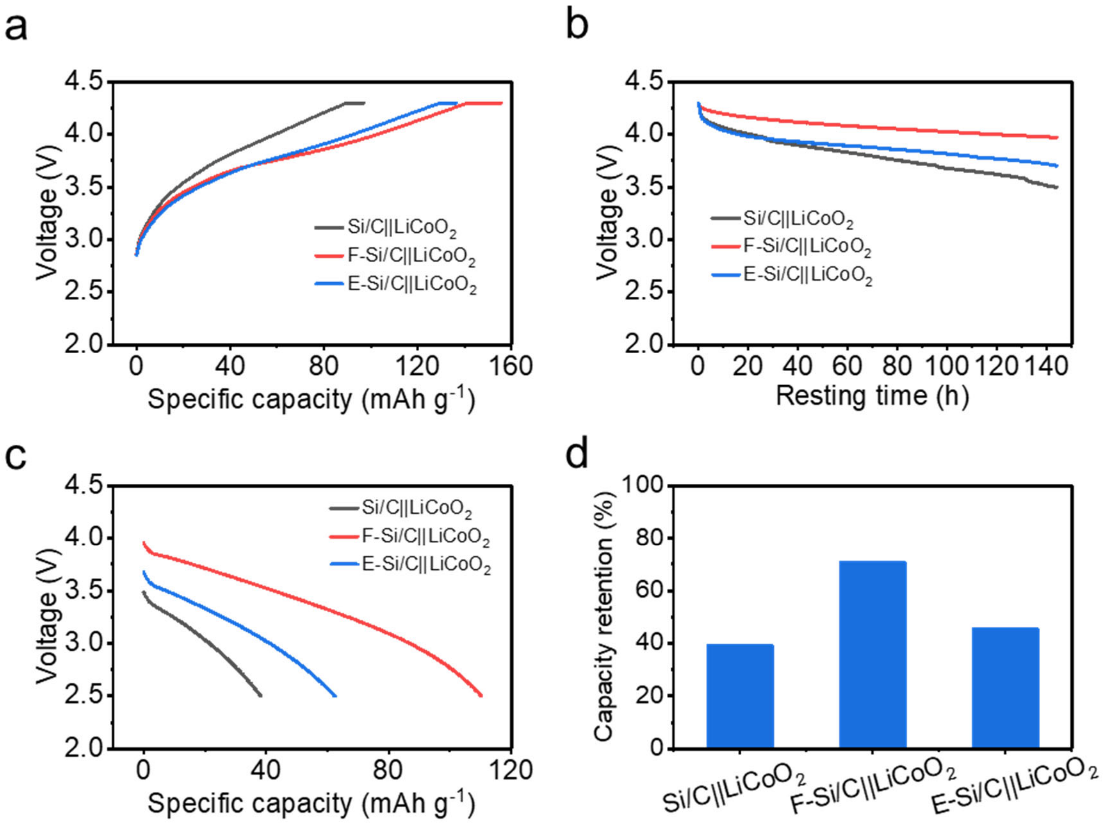


**Supplementary Fig. 21 | High-temperature storage performance for Si/C||$LiCoO_2$, Si/C||F-$LiCoO_2$ and Si/C||E-$LiCoO_2$ cells.** The charge profiles before exposure to high temperature (a), the OCV during storage at 50 °C for 6 days (b) and the discharge profiles at 25 °C (c) of the Si/C||$LiCoO_2$, Si/C||F-$LiCoO_2$ and Si/C||E-$LiCoO_2$ cells, and the corresponding comparison capacity retention (d).

High-temperature storage performance tests for different electrodes were conducted at 55 °C. Prior to the measurement, the Si/C||$LiCoO_2$, Si/C||F-$LiCoO_2$ and Si/C||E-$LiCoO_2$ cells were subjected to a pre-cycling process (with a potential range of 4.3-2.5 V at 0.1 C, at 25 °C), which facilitated the formation of the SEI. Subsequently, all the cells were charged to a voltage of 4.3 V at 0.5 C and subjected to a storage period of 6 days at 55 °C. Following this storage period, the cells were allowed to rest at 25 °C and discharged to a voltage of 2.5 V in order to measure the corresponding capacity values, denoted as “Retained capacity”, which was utilized to evaluate the performance of the cells during high-temperature storage. When stored at 55 °C, the OCV of the F-

$Si/C||LiCoO_2$ battery was more stable, and its retained capacity and capacity retention rate (110.3 mAh $g^{-1}$ and 70.9% respectively) were higher than those of the $LiCoO_2||Si/C$ battery (38.3 mAh $g^{-1}$ and 39.4% respectively) and the $LiCoO_2||E\text{-}Si/C$ battery (62.4 mAh $g^{-1}$ and 45.7% respectively). Therefore, the $LiCoO_2||F\text{-}Si/C$ battery exhibited better high-temperature storage performance than the counterparts.

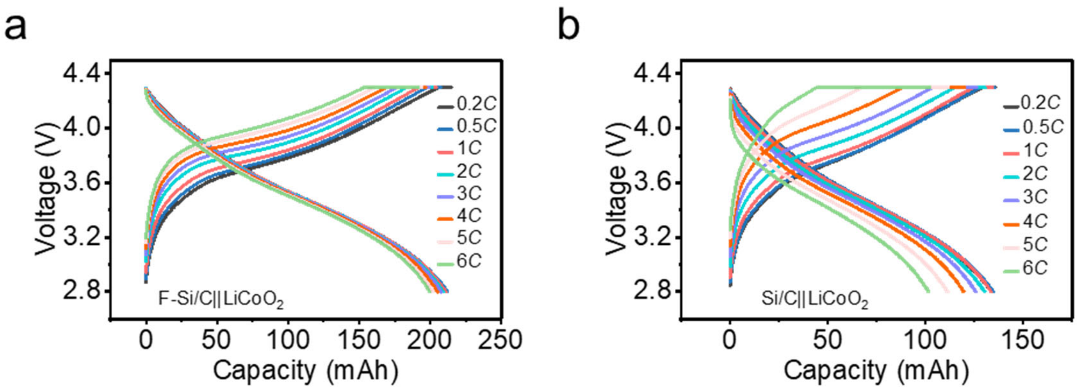


**Supplementary Fig. 22 | The voltage profiles of the F-Si/C||$LiCoO_2$ (a) and Si/C||$LiCoO_2$ (b) cells at various charging rates. The discharging current was maintained at 0.2C.**

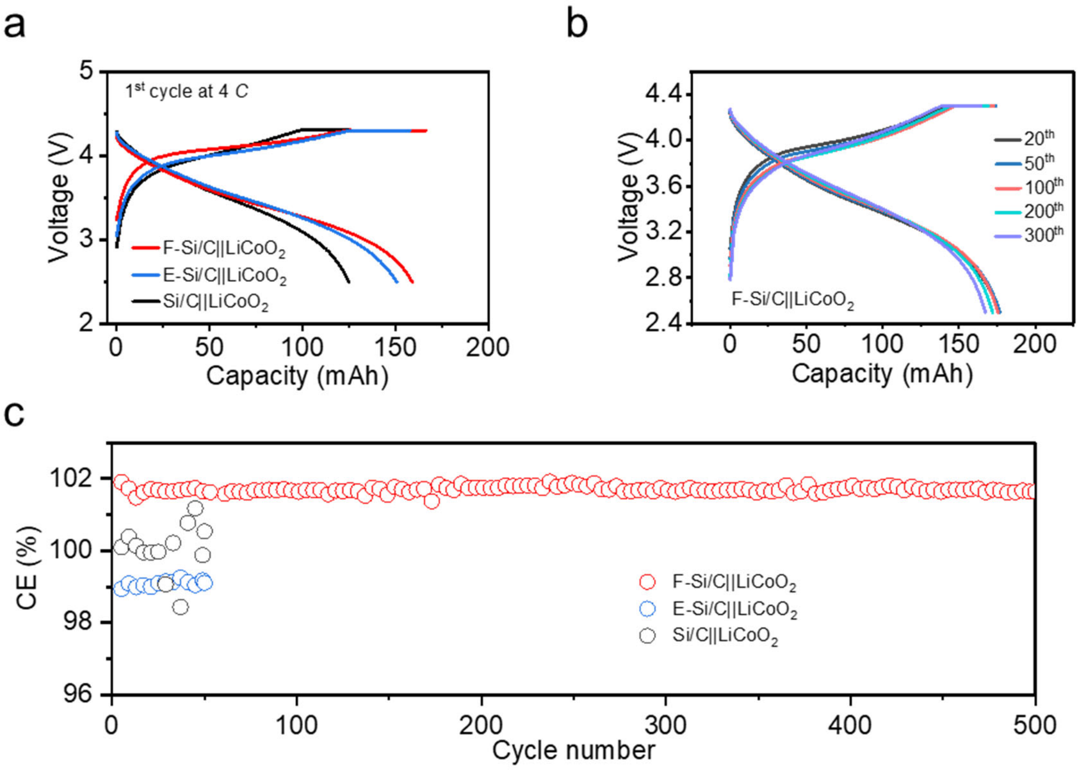


**Supplementary Fig. 23 | a, The 1st-cycle voltage profiles of the F-Si/C||$LiCoO_2$, E-Si/C||$LiCoO_2$ and Si/C||$LiCoO_2$ pouch cells at 4C. b, The voltage profiles of the F-Si/C||$LiCoO_2$ cell at different cycles. c, The cyclic CE of the F-Si/C||$LiCoO_2$, E-Si/C||$LiCoO_2$ and Si/C||$LiCoO_2$ pouch cells.**

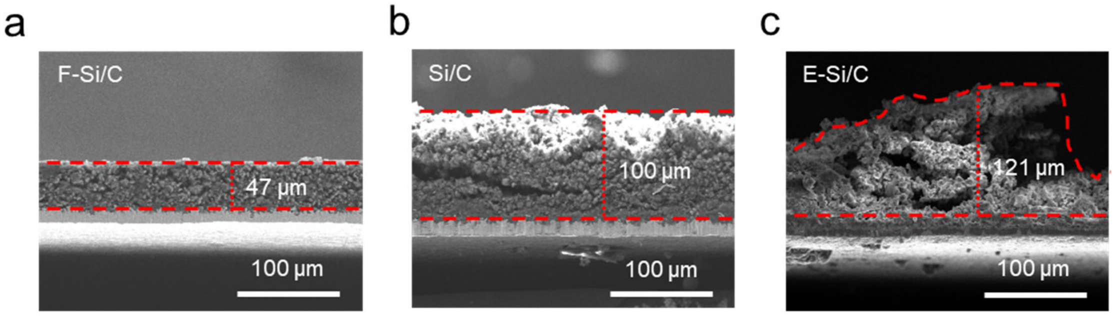


**Supplementary Fig. 24 | Cross-sectional SEM images of the F-Si/C (a), Si/C (b), and E-Si/C (c) electrodes after 100 cycles.**

The SEM image revealed the thickness of different Si/C electrodes after cycling. The thickness of the F-Si/C electrode increased from 38 μm to 47 μm. The pristine Si/C electrode exhibited an increase from 24 μm to 100 μm, while the E-Si/C electrode showed an increase from 55 μm to 121 μm. The corresponding expansion ratios were calculated as 24%, 317%, and 120%, respectively. Notably, the F-Si/C electrode demonstrated the smallest expansion ratio among these electrodes, further confirming its structural stability.

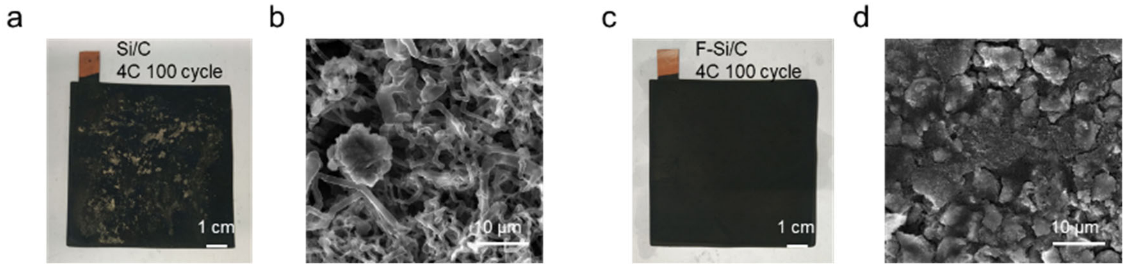


**Supplementary Fig. 25 | Optical photograph and SEM image of Si/C (a, b) and F-Si/C (c, d) electrodes after 100 cycles with a charging current of 4C.**

The morphology and structure of the F-Si/C electrode and pristine Si/C electrode after cycling in pouch cell under fast charging were investigated. The digital and SEM images reveal noteworthy observations following 100 cycles at 4C. The cycled pristine Si/C electrode exhibited substantial silvery-grey deposits (Supplementary Fig. 25a). In stark contrast, the cycled F-Si/C electrode displayed a conspicuous absence of such deposits (Supplementary Fig. 25c). Examination of the SEM images provided further insights into the nature of the electrodes. Supplementary Fig. 25b illustrates the presence of dendritic and mossy metallic Li structures on the protrusions of the cycled pristine Si/C electrode. In contrast, the SEM image of the cycled F-Si/C electrode (Supplementary Fig. 25d) showcases a smooth and clean surface devoid of any Li deposits. The suppressed Li plating behavior of the cycled F-Si/C electrode can be attributed to the enhanced $Li^+$ transport capability of the F-Si/C electrode, which significantly improve the fast-charging performance.

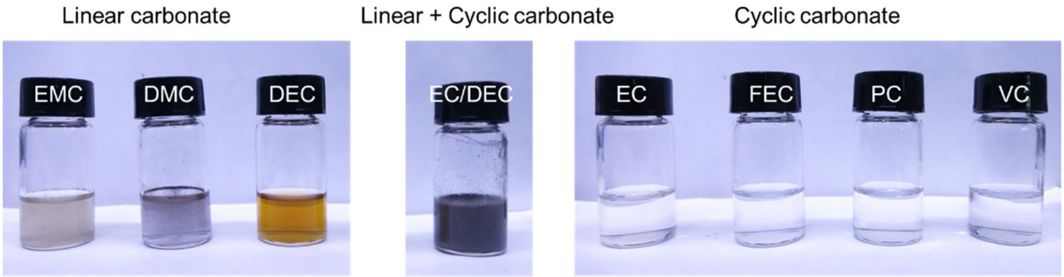


**Supplementary Fig. 26 | Optical images of solvents after their reactions with metallic Li by resting at 40 °C for 7 days (10 mg metallic Li in 4 mL solvent).**

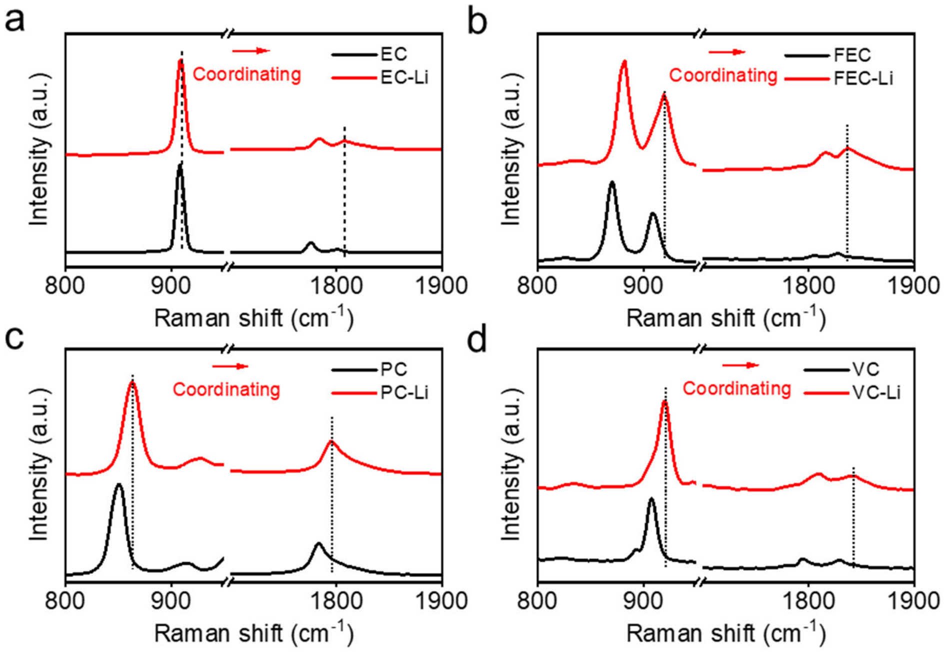


**Supplementary Fig. 27 | Raman spectra of the cyclic carbonate-Li solutions. a-d,** Raman spectra of the EC-Li solutions (**a**), FEC-Li solutions (**b**), PC-Li solutions (**c**) and VC-Li solutions (**d**).

Characteristic peaks of the pristine cyclic carbonate solvents (EC, FEC, PC and VC) were predominantly observed in the regions of 800–900 and 1800–1900 $cm^{-1}$. By comparison, characteristic peaks of the cyclic carbonate-Li solutions showed a blue shift, suggesting the existence of $Li^+$ solvation structures.

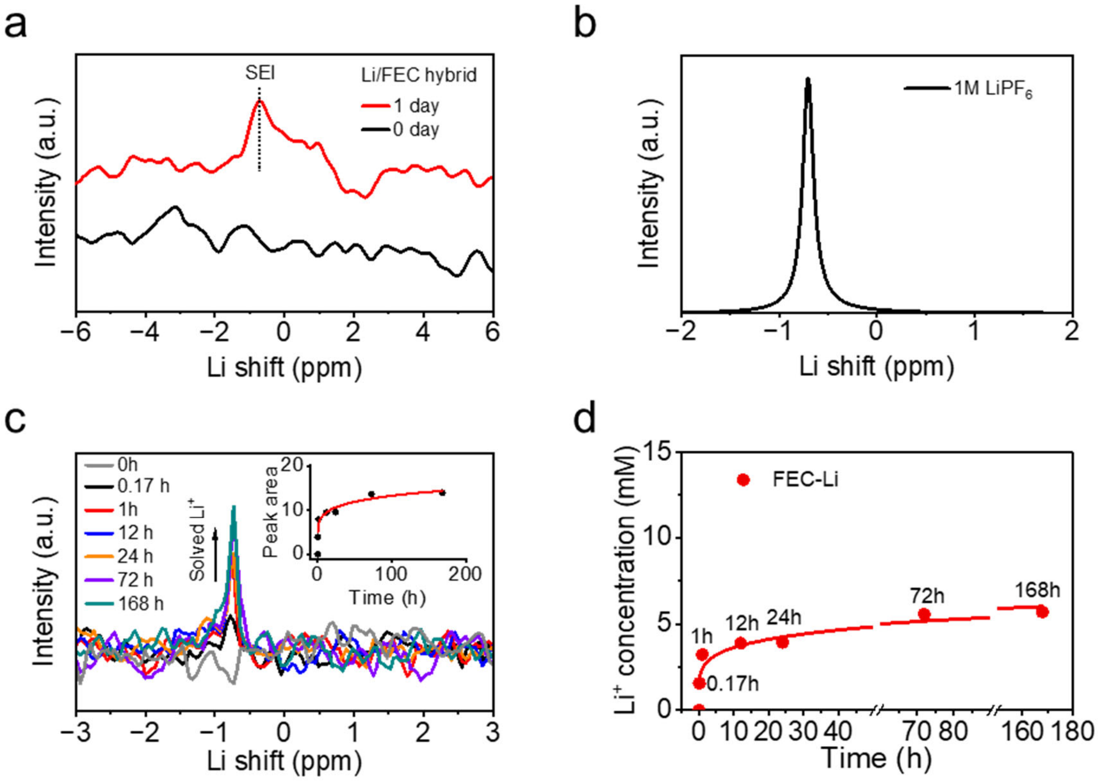


**Supplementary Fig. 28 | NMR spectra of the FEC solvent after their reactions with metallic Li. a**, $^7$Li solid-state NMR (SSNMR) spectra of metallic Li and FEC hybrid for different times. **b**, $^7$Li NMR spectrum of the regular electrolyte (1 M $LiPF_6$ in EC/DEC, v/v = 1/1). **c**, $^7$Li NMR spectra of the FEC solvent reacted with metallic Li during 7 days and the corresponding $Li^+$ peak area-time plot. **d**, $Li^+$ concentration-time plot of the FEC-Li solution with a duration of 7 days and the corresponding $^7$Li NMR spectra.

SSNMR spectroscopy of the FEC and Li metal hybrid was acquired to characterize the reaction between Li metal and FEC (Supplementary Fig. 28a). There appeared a new $^7$Li NMR signal centered around 0 ppm corresponding to characteristics of Li species from SEI, suggesting the occurrence of the reaction between the Li metal and FEC[3]. The evolution of $Li^+$ ions was monitored by the $^7$Li NMR spectra of the FEC-Li solution after different resting times. The result demonstrated the quick emergence of a peak centered at~0 ppm after 0.17 h, and the signal maintained good stability in intensity with only a slight increase during resting over the test time (168 h). The values of Li

content were calculated based on the peak area using Li content in the regular electrolyte (1 M $LiPF_6$ in EC/DEC, v/v = 1/1) as the reference (Supplementary Figs. 28b-d). The value of the calculated $Li^+$ concentration was at the mmol level, which could be explained by the passivation of the Li metal surface by solid electrolyte interphase (SEI).

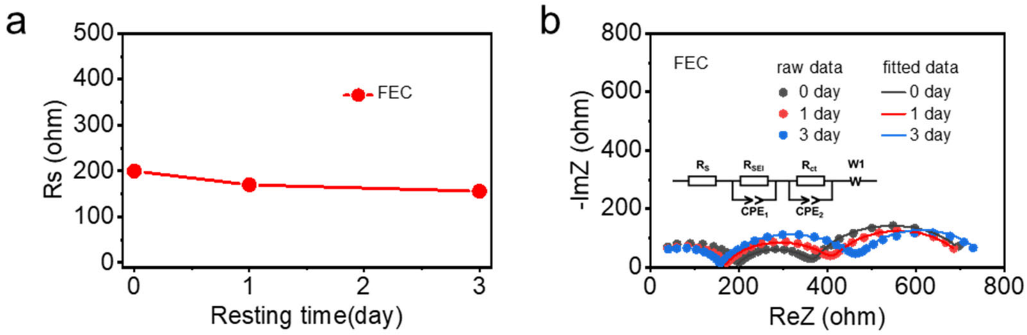


**Supplementary Fig. 29 | The evolution of resistance for Li||Li symmetric cells in FEC solvent. a**, EIS results of Li||Li symmetric cells with different resting times in FEC solvent. **b**, Nyquist plots of Li||Li symmetric cells with FEC solvent.

It was observed that the series resistance term ($R_s$) of cells with FEC remained stable (~200 Ω) with the resting time from 0 to 3 days, showing the good stability of the Li-FEC solution.

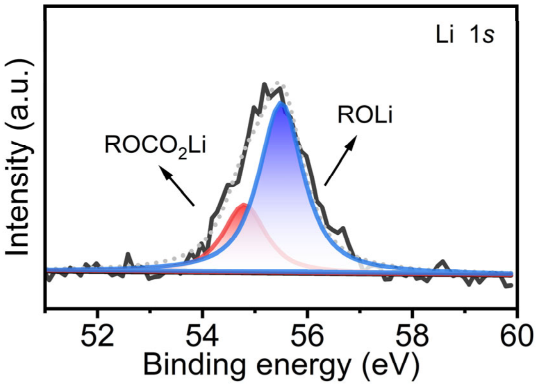


**Supplementary Fig. 30 | High-resolution Li 1*s* XPS spectrum of the metallic Li after resting in FEC solvent for 7 days.**

High-resolution Li 1*s* XPS spectrum for the metallic Li after its resting in FEC solvent showed two peaks at 54.8 and 55.5 eV corresponding to $ROCO_2Li$ and ROLi, respectively, which verified the formation of organic Li alkyl carbonate components on Li metal surface[4]. The formation of such an interphase (SEI) explains that a continuous reaction between metallic Li and FEC would not take place, which helps to maintain a stable $Li^+$ concentration in FEC over extended resting times. The passivation of the metallic Li surface accounts for the low and stable $Li^+$ concentration in FEC, which could facilitate a warm, uniform prelithiation reaction.

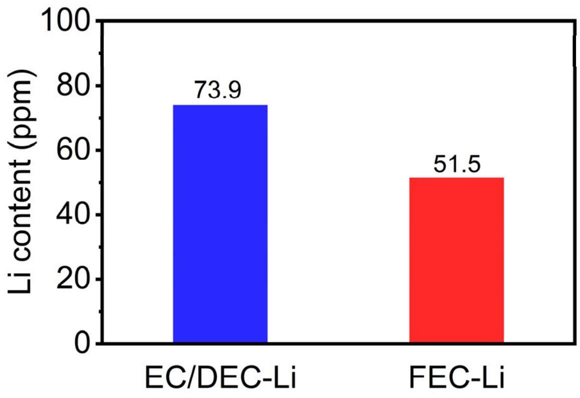


**Supplementary Fig. 31 | Li contents of carbonate-Li solutions measured by ICP-OES.**

The EC/DEC-Li and FEC-Li solutions showed low Li content of 73.9 and 51.5 ppm and the corresponding values of $Li^+$ concentration was 0.01 and 0.007 M. This result suggests that cyclic carbonates could react with metallic Li to produce the corresponding dissolved Li alkyl carbonate species with ultralow $Li^+$ concentration.

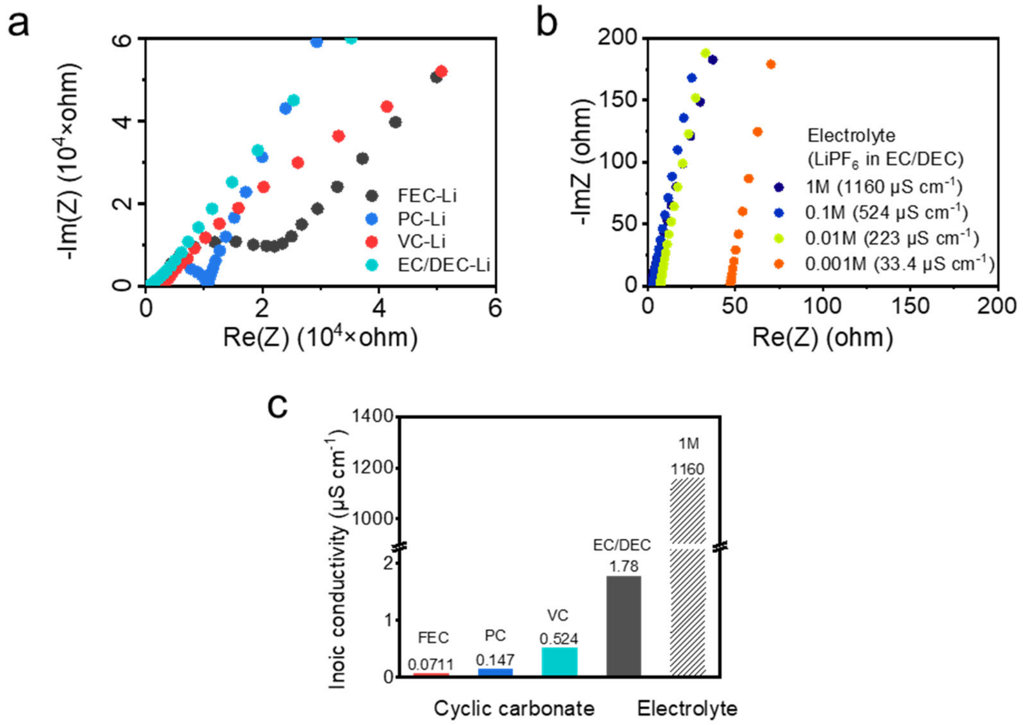


**Supplementary Fig. 32 | Nyquist plots of (a) different cyclic carbonate-based solvents after their reactions with metallic Li and (b) electrolytes with different $Li^+$ concentrations.** Nyquist plots was obtained by assembled spacer||PP||spacer cells with carbonate-Li solution or electrolyte. **c**, Comparison of ionic conductivity for different solvent-Li solutions.

Cyclic carbonate-Li solutions displayed low values of ionic conductivity (0.0711, 0.147, 0.524 and 1.78 µS $cm^{-1}$ for FEC-Li, PC-Li, VC-Li and EC/DEC-Li, respectively). These values were close to the ultralow Li salt concentration electrolyte (e.g., 33.4 µS $cm^{-1}$, 1 mM $LiPF_6$ in EC/DEC) and significantly lower than the regular electrolyte (~1 mS $cm^{-1}$, 1 M $LiPF_6$ in EC/DEC).

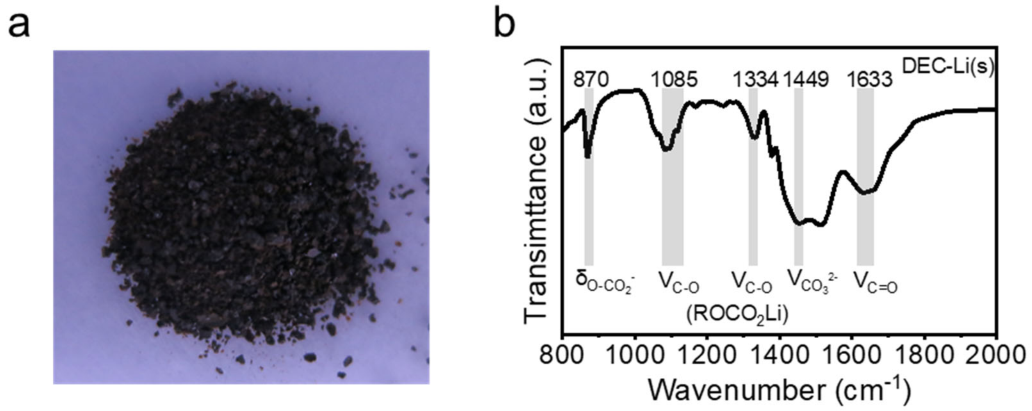


**Supplementary Fig. 33 | The investigation of the solid product after the reaction between metallic Li and DEC. a**, **b**, Digital image (**a**) and FTIR spectra (**b**) of the solid product between metallic Li and DEC (namely, solid DEC-Li). Metallic Li reacts with DEC to produce a solid product and can be easily separated from the solvent, which is composed of Li alkyl carbonate species.

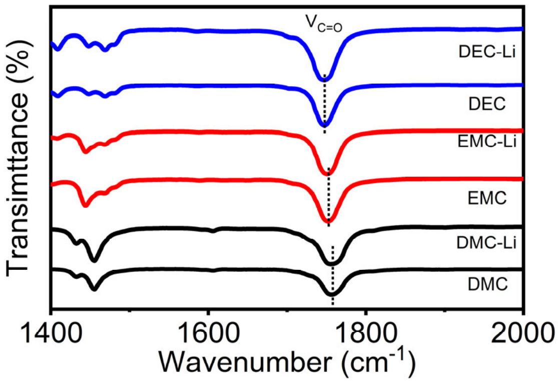


**Supplementary Fig. 34 | FTIR spectra of the linear carbonate-Li solutions.**

As shown in Supplementary Fig. 34, The FTIR spectra of the linear carbonate-Li solutions showed no shift in the vibration peak of C=O for the DEC-Li, dimethyl carbonate-Li (DMC-Li) and ethyl methyl carbonate-Li (EMC-Li) solutions compared with the corresponding pure solvents, suggesting no observable coordination between $Li^+$ ions and solvents. This result supports that the product from the reaction between metallic Li and these linear carbonates could not be effectively dissolved.

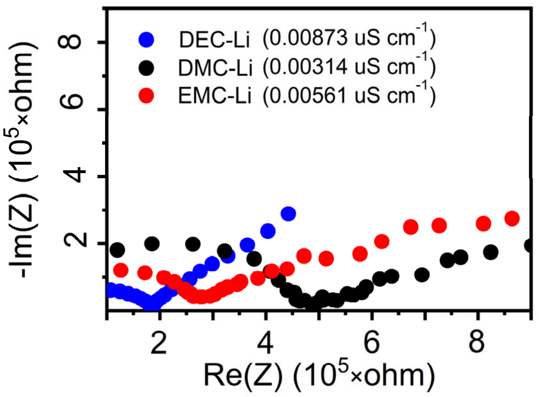


**Supplementary Fig. 35 | Nyquist plots and ionic conductivity results of different linear carbonate solvents after their reactions with metallic Li.**

Linear carbonate-Li solutions displayed ultralow values of ionic conductivity (0.00873, 0.00314 and 0.00561 μS $cm^{-1}$ for DEC-Li, DMC-Li and EMC-Li, respectively), which were significantly lower than the cyclic carbonate-Li solution (1.78 μS $cm^{-1}$ for the EC/DEC-Li solution). This result excludes their function as mediums for contact prelithiation due to their ultralow solubility.

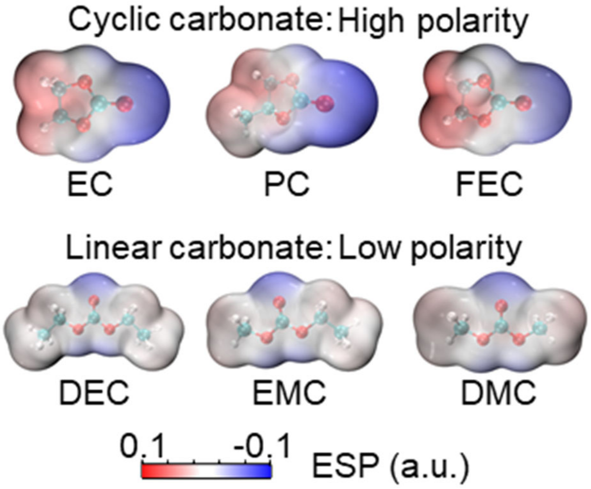


**Supplementary Fig. 36 | Calculated electrostatic potential maps of carbonate solvents.**

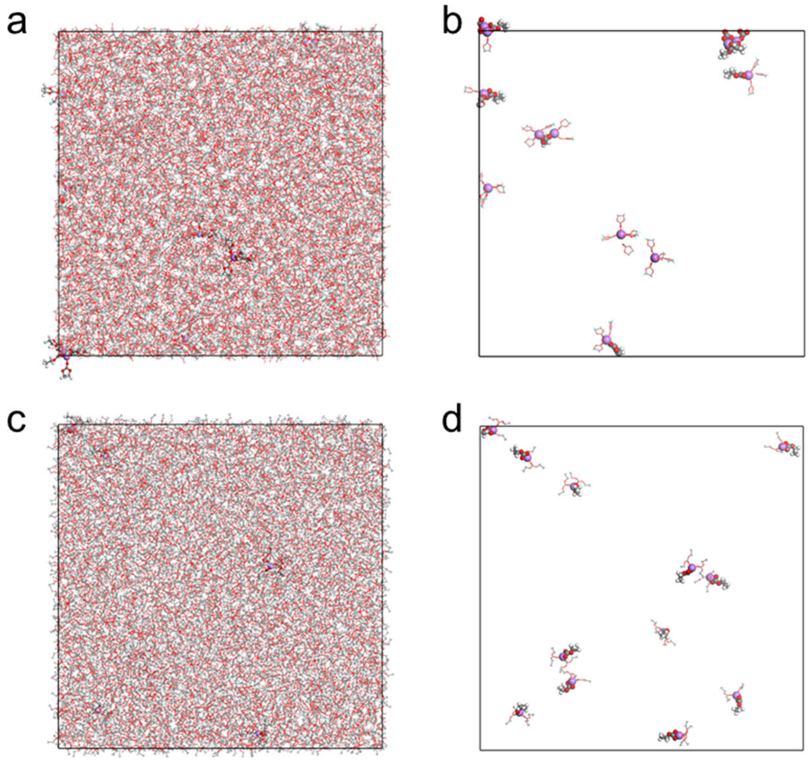


**Supplementary Fig. 37 | MD simulations using Li ethyl carbonate (LiEC). a-d,** MD snapshot of LiEC dissolving in FEC (**a**, **b**) and DEC (**c**, **d**). The Li-ions and $EC^-$ ions are represented by the CPK scale.

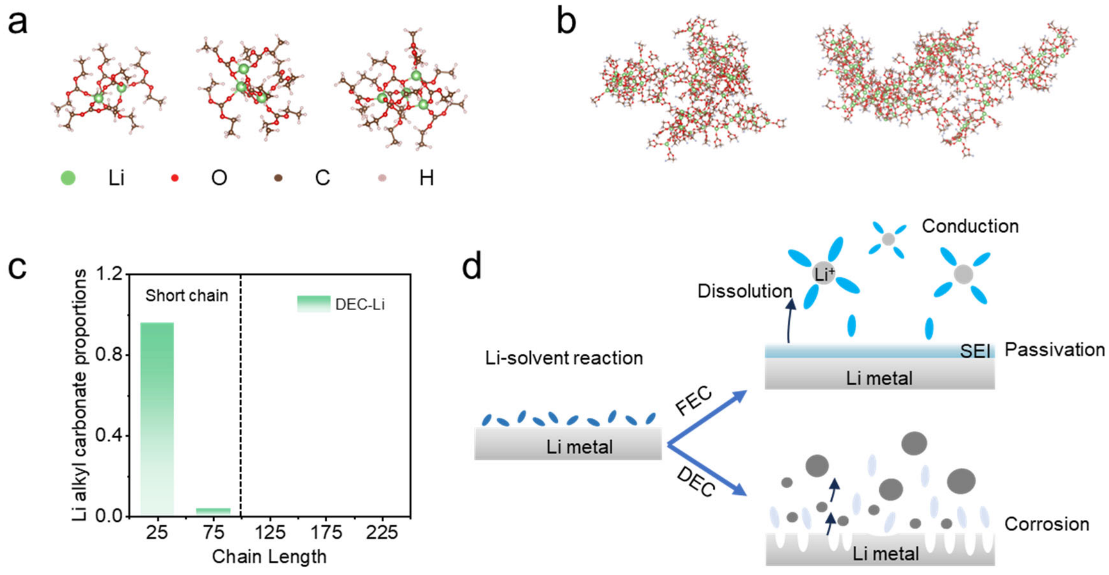


**Supplementary Fig. 38 | Calculations of the structure of Li alkyl carbonates produced via the carbonate solvent-Li reaction.** **a**, Typical short chain cluster structures of LEDC in DEC solvent. **b**, Longer chain structures of LFEDC in FEC solvent. **c**, Calculations of the structure of Li alkyl carbonates in DEC solvent. **d**, Schematic representation of Li metal in different solvents.

As shown in Supplementary Fig. 38, our calculations reveal that short-chain Li alkyl carbonates tend to form in the linear DEC solvent, indicating that a stable SEI is not formed and results in an ongoing reaction. In contrast, long-chain Li alkyl carbonates are capable of forming an extensive polymer network in FEC solvent and participating in the formation of a stable SEI on the Li metal surface to suppress the continuous Li-solvent reaction.

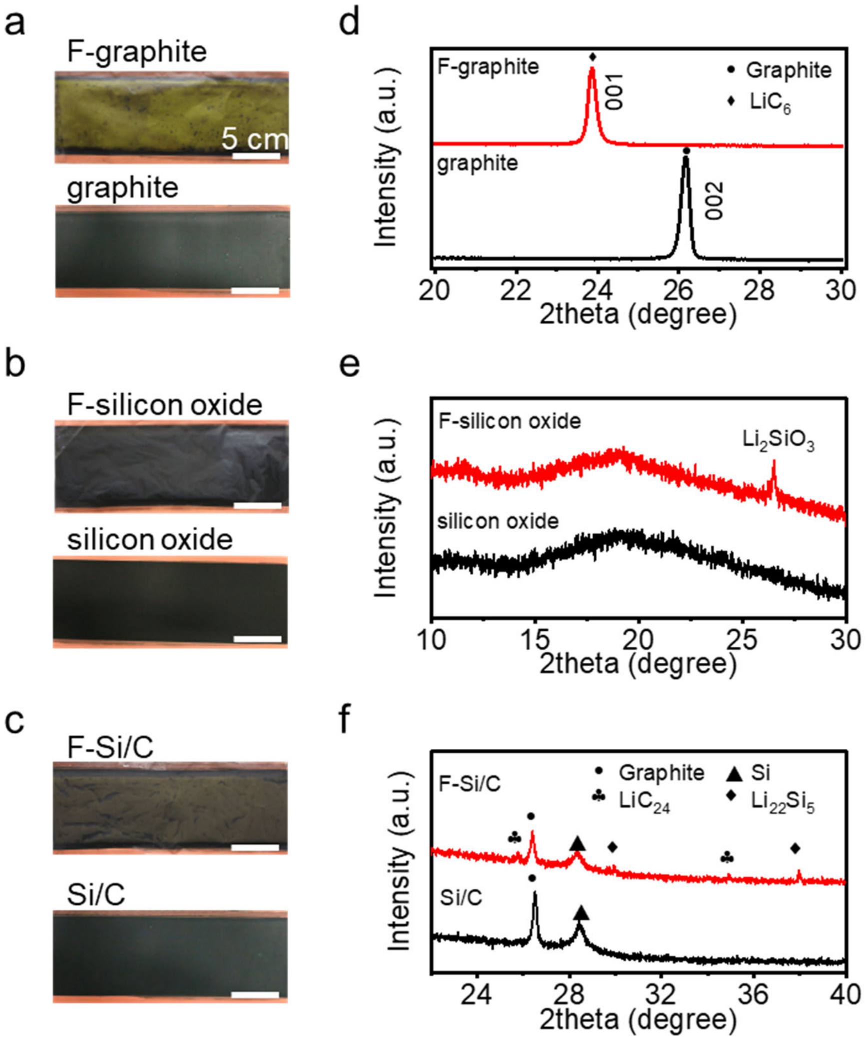


**Supplementary Fig. 39 | Characterizations of different anodes after contact prelithiation**. **a-c**, Photograph of the graphite and F-graphite electrodes (**a**), the silicon oxide and F-silicon oxide (**b**) and the Si/C and F-Si/C (**c**) electrodes. **d-f**, The corresponding XRD patterns of the graphite and F-graphite electrodes (**d**), the silicon oxide and F-silicon oxide electrodes (**e**) and the Si/C and F-Si/C (**f**) electrodes.

The graphite, silicon oxide and Si/C electrodes were deeply prelithiated via an as-developed contact prelithiation approach using FEC medium (Supplementary Figs. 39a-c). The color change was shown for all the electrodes after FM-prelithiation. Typically, the graphite electrode with the size of 8 x 25 cm turned from grey to gold after prelithiation. The structure and phase change of the graphite and silicon oxide electrodes before and after FM-prelithiation were measured by XRD (Supplementary Figs. 39d-f). The typical (002) peak at 26° for the C (graphite) phase was shifted to

24.0° after the prelithiation of the graphite electrode, which corresponded to the (001) peak of the $LiC_6$ phase. A new XRD peak at 27.0° emerged after the prelithiation of silicon oxide, which was recognized as $Li_2SiO_3$. The Si/C electrode was successfully prelithiated, as confirmed by the formation of $Li_{22}Si_5$ and $LiC_{24}$ phases in the XRD pattern. These results verified the successful prelithiation of all the three anodes using the FEC medium.

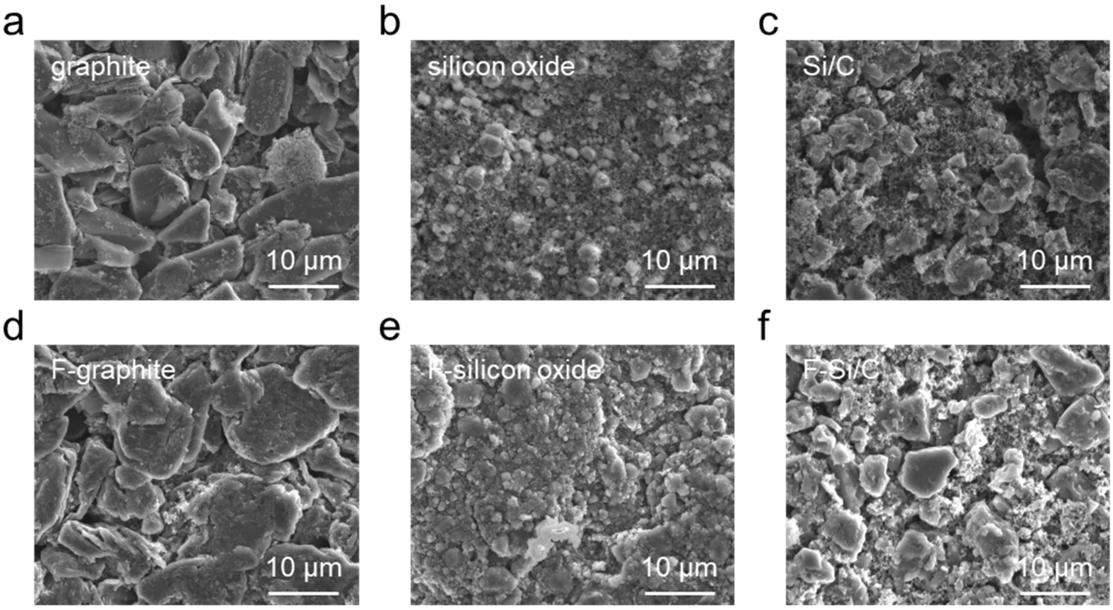


**Supplementary Fig. 40 | SEM images of various prelithiated anodes using FEC medium. a-f,** SEM images of the graphite (**a**), silicon oxide (**b**), Si/C (**c**), F-graphite (**d**), F-silicon oxide (**e**) and F-Si/C (**f**) electrodes. As evidenced by the top-view SEM images, all the prelithiated electrodes, including F-graphite, F-silicon oxide and F-Si/C electrodes, effectively preserved their initial morphology and configuration without damage.

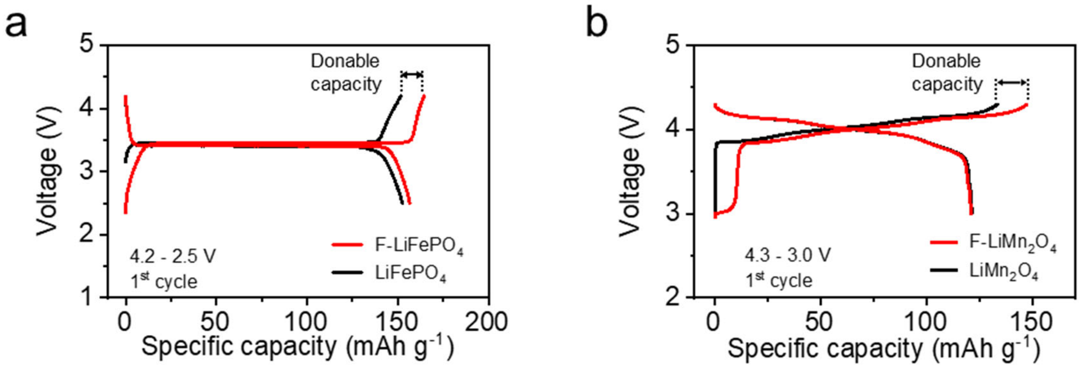


**Supplementary Fig. 41 | The first-cycle voltage profiles of the $LiFeO_4$ and F-$LiFeO_4$ electrodes at a specific current of 0.015 A $g^{-1}$ (a) and $LiMn_2O_4$ and F-$LiMn_2O_4$ (b) electrodes at a specific current of 0.01 A $g^{-1}$.**

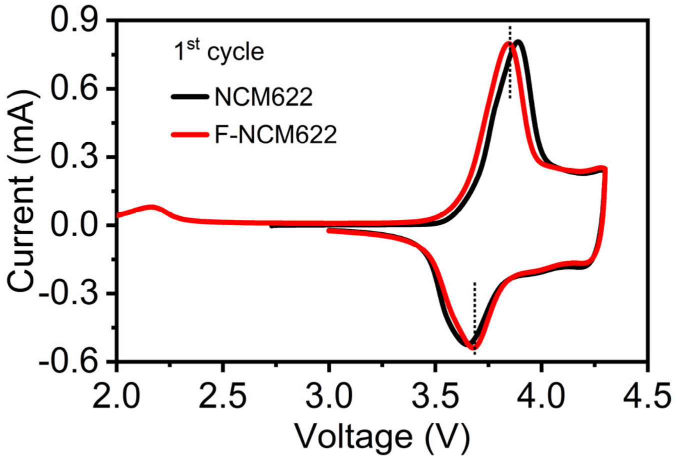


**Supplementary Fig. 42 | The initial CV curves of the NCM622 and F-NCM622 electrodes in the potential range of 3.0–4.3 V at a scan rate of 0.05 mV $s^{-1}$.**

The F-NCM622 showed highly overlapped CV curves with a cathodic peak at 3.84 V and an anodic peak at 3.68 V, whose location and peak intensity were very close to those of the pristine NCM622. This result supports the great advantage of the solvent-mediated prelithiation reaction for achieving good electrochemical stability.

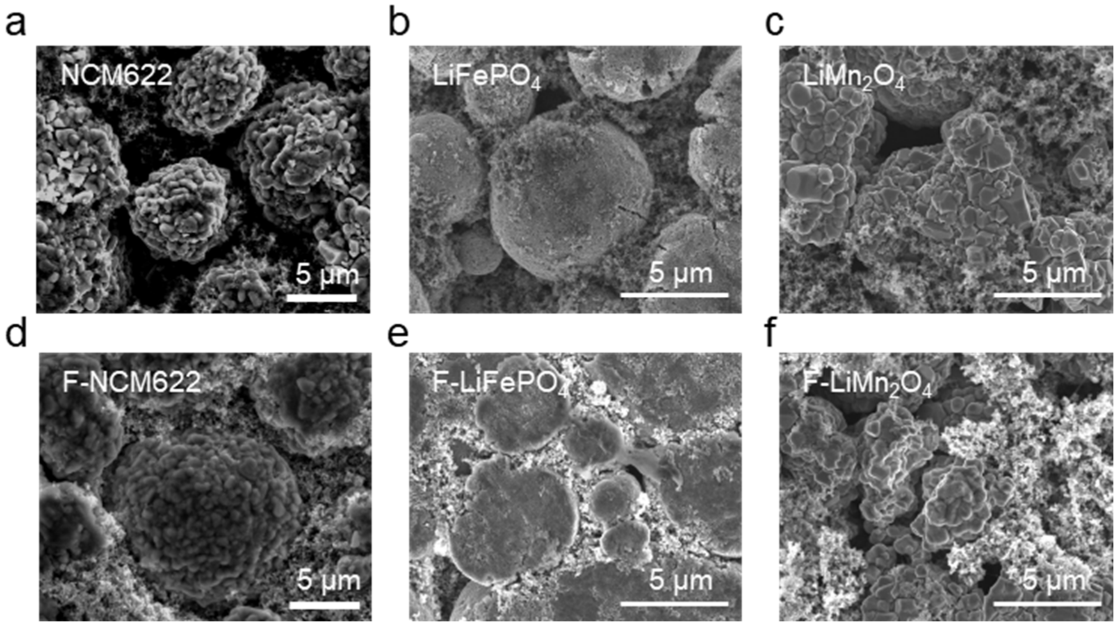


**Supplementary Fig. 43 | SEM images of the NCM622 (a), $LiFePO_4$ (b) and $LiMn_2O_4$ (c), F-NCM622 (d), F-$LiFePO_4$ (e) and F-$LiMn_2O_4$ (f) electrodes.**

The results of the SEM investigation on the F-NCM622, F-$LiFePO_4$ and F-$LiMn_2O_4$ revealed that all three cathodes/active particles maintained their initial structures without showing any cracks at both electrode and particle levels.

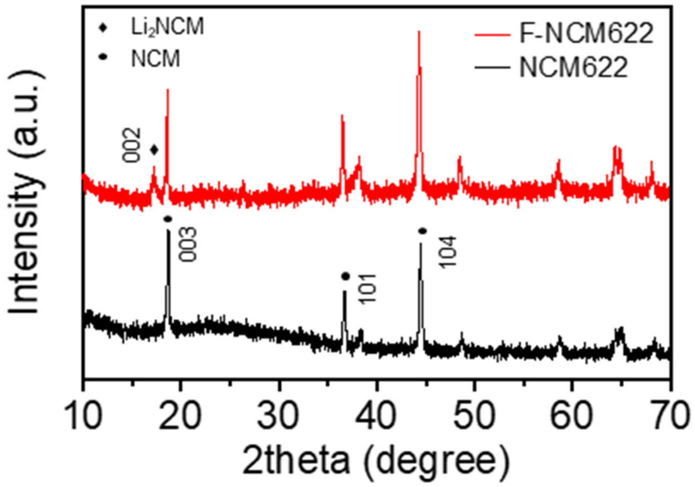


**Supplementary Fig. 44 | XRD patterns of the NCM622 and F-NCM622 electrodes.** In comparison to the peaks for the pristine NCM622 phase with the R-3m space group, a new characteristic XRD peak at 17.5° appeared for the F-NCM622, corresponding to the $Li_2$NCM phase with the P3m1 space group, suggesting its successful prelithiation[5].

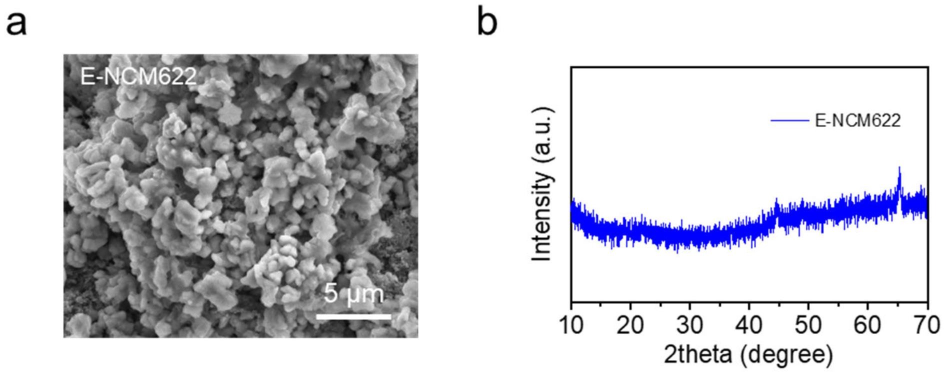


**Supplementary Fig. 45 | SEM image (a) and XRD pattern (b) of the NCM622 electrode after electrolyte-mediated prelithiation (E-NCM622) electrodes.**

Supplementary Fig. 45a showed the SEM image of the E-NCM622 electrode. Compared with the pristine NCM622 (Supplementary Figure 43a), it was clearly observed that the particles were broken. The E-NCM622 displayed a notable decrease in the XRD characteristic peak intensity in comparison to the pristine counterpart (Supplementary Fig. 45b and Fig. 44). These findings suggested that prelithiation through electrolyte-mediated contact prelithiation resulted in non-uniform prelithiation products and compromises the crystalline structure of NCM622. Conversely, the results presented above highlight the significant advantage of FEC-mediated prelithiation in preserving the structural integrity of the material.

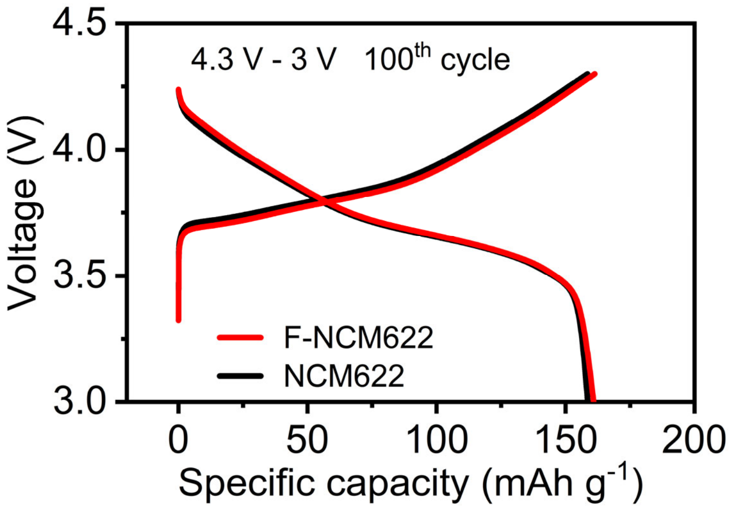


**Supplementary Fig. 46 | The voltage profiles of NCM622 and F-NCM622 electrodes for the 100th cycle at a specific current of 0.1 A $g^{-1}$.**

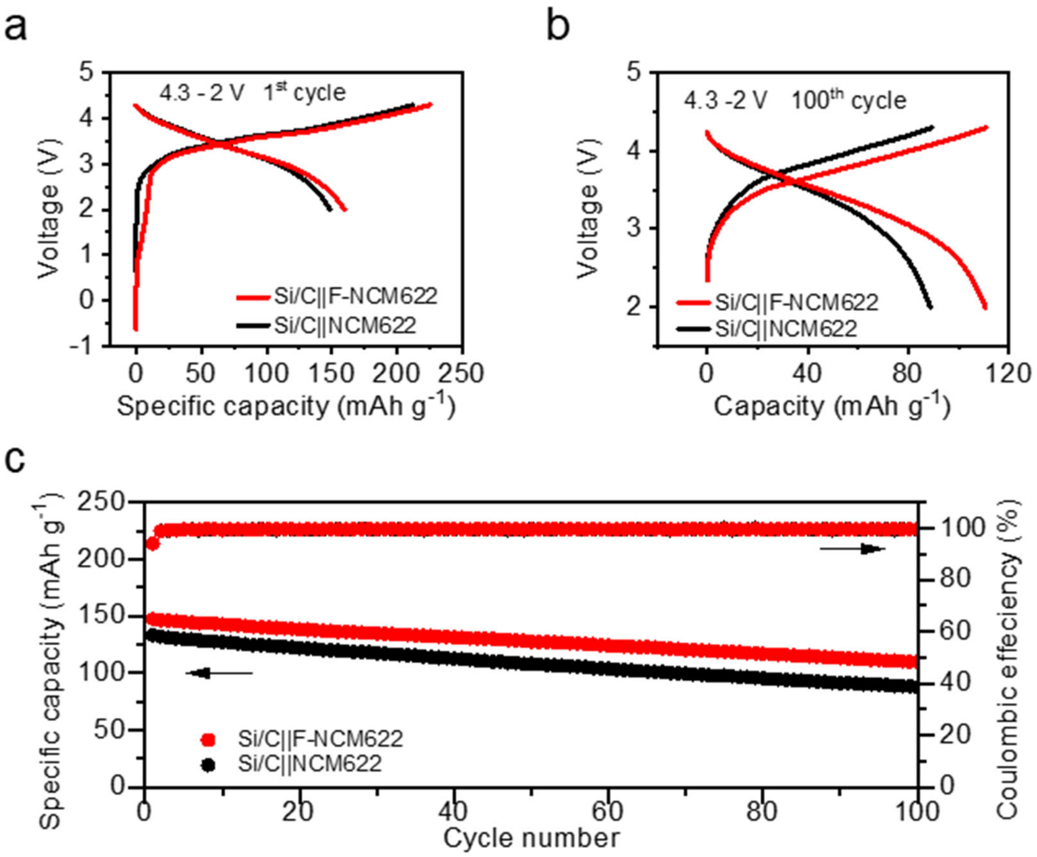


**Supplementary Fig. 47 | The electrochemical performance of Si/C||NCM622 and Si/C||F-NCM622 cells. a-c**, The first-cycle voltage profiles at a specific current of 0.02 A $g^{-1}$ (**a**) and the 100$^{th}$-cycle voltage profiles at a specific current of 0.1 A $g^{-1}$ (**b**) and the corresponding cycling performance at a specific current of 0.1 A $g^{-1}$ (**c**).

The Si/C||F-NCM622 cells delivered a reasonably higher capacity of 13 mAh $g^{-1}$ in comparison to the Si/C||NCM622 cells during the first cycle, and higher capacity retention for 100 cycles in comparison to the cell with a pristine NCM622 electrode.

**Supplementary Table 1.** Discharge capacities of the Si/C electrodes after prelithiation using electrolytes with various $Li^+$ concentrations.

| | **Time (min)** | **Discharge capacity (mAh $g^{-1}$)** | | |
|---|---|---|---|---|
| **1M** | **0.33** | 1528 | 1534 | 1581 |
| | **1** | 1432 | 1406 | 1437 |
| | **2** | 1293 | 1375 | 1375 |
| | **3** | 1210 | 1235 | 1329 |
| | **6** | 966 | 884 | 1117 |
| **0.1M** | **1** | 1554 | 1596 | 1572 |
| | **5** | 1460 | 1479 | 1430 |
| | **10** | 1281 | 1330 | 1228 |
| | **20** | 973 | 971 | 1044 |
| | **30** | 719 | 685 | 743 |
| **0.01M** | **10** | 1548 | 1582 | 1608 |
| | **30** | 1326 | 1384 | 1366 |
| | **40** | 1254 | 1245 | 1303 |
| | **60** | 1069 | 1099 | 1142 |
| | **80** | 946 | 976 | 995 |
| **0.001M** | **10** | 1689 | 1667 | 1667 |
| | **30** | 1615 | 1648 | 1637 |
| | **60** | 1590 | 1568 | 1597 |
| | **120** | 1390 | 1401 | 1463 |
| | **240** | 1215 | 1225 | 1242 |
| | **300** | 972 | 969 | 1053 |

**Supplementary Table 2.** EIS fitting results for Si/C, F-Si/C and E-Si/C electrodes at different temperatures.

| Sample | Temperature (°C) | $R_S$ | $R_{SEI}$ | $R_{CT}$ |
|---|---|---|---|---|
| **Si/C** | 10 | 3 | 25 | 172 |
| | 15 | 3 | 40 | 119 |
| | 20 | 2 | 33 | 77 |
| | 30 | 3 | 2 | 30 |
| | 40 | 3 | 9 | 13 |
| | 50 | 3 | 2 | 7 |
| **F-Si/C** | 10 | 2 | 20 | 84 |
| | 15 | 2 | 13 | 60 |
| | 20 | 3 | 6 | 38 |
| | 30 | 3 | 3 | 22 |
| | 40 | 1 | 2 | 9 |
| | 50 | 3 | 1.7 | 5 |
| **E-Si/C** | 10 | 2 | 27 | 198 |
| | 15 | 3 | 30 | 139 |
| | 20 | 2 | 14 | 88 |
| | 30 | 3 | 2 | 36 |
| | 40 | 3 | 3 | 16 |
| | 50 | 3 | 2 | 8 |

**Supplementary Table 3.** The detailed parameters of the fabricated Ah-level laminated pouch cells.

| Cell parameters of the pouch cell | | | | | |
|---|---|---|---|---|---|
| | **Parameter** | **Value** | | **Parameter** | **Value** |
| **Cathode** | Specific capacity | 180 mAh $g^{-1}$ | **Anode** | Specific capacity | 1200 mAh $g^{-1}$ |
| | Area weight (each side) | 11.5 mg $cm^{-1}$ | | Area weight (each side) | 2 mg $cm^{-1}$ |
| | Thickness | 40 µm | | Thickness | 20 µm |
| | Number of layers | 11 | | N/P ratio | 1.16 |

**Supplementary Table 4.** The calculated results of the specific capacity and energy density of the Ah-level $LiCoO_2$||F-Si/C and $LiCoO_2$||Si/C pouch cells at different *C* rates (based on the total mass of cathode and anode, excluding the mass of the current collector).

| | **$LiCoO_2$\|\|F-Si/C pouch cell** | | **$LiCoO_2$\|\|Si/C pouch cell** | |
|---|---|---|---|---|
| ***C*-rate** | Specific Capacity (mAh $g^{-1}$) | Energy Density (W h $kg^{-1}$) | Specific Capacity (mAh $g^{-1}$) | Energy Density (W h $kg^{-1}$) |
| **0.1** | 169.3 | 464.6 | 119.8 | 325.7 |
| **1** | 144.9 | 395.4 | 105.6 | 273.2 |

**Supplementary Table 5.** Comparison with Si-based cell (2021-2024).

| Current density | Cycle number | Capacity retention rate (%) | Cell types | Anode capacity (mAh $g^{-1}$) | Energy density (W h $kg^{-1}$) | Ref. |
|---|---|---|---|---|---|---|
| **1 *C*** | **350** | **93** | **Ah-level pouch cell** | **1200** | **464.6** | **This work** |
| **4 *C*** | **400** | **90** | **pouch cell** | **1200** | **406.1** | **This work** |
| **10 *C*** | **100** | **95.3** | **pouch cell** | **1200** | **436.7** | **This work** |
| 4 *C* | 250 | 83.8 | Ah-level pouch cell | 1050 | 410 | Ref. 6 |
| 3 *C* | 700 | 81.3 | Ah-level pouch cell | 1912 | - | Ref. 7 |
| 2 *C* | 450 | 80.9 | Ah-level Pouch cell | 650 | 300 | Ref. 8 |
| 1 *C* | 400 | 87.2 | Ah-level pouch cell | 500 | - | Ref. 9 |
| 1 *C* | 500 | 80.3 | Ah-level pouch cell | 500 | - | Ref. 10 |
| 1 *C* | 700 | 80.2 | Ah-level pouch cell | 500 | 270.2 | Ref. 11 |
| - | 180 | 65.4 | Ah-level Pouch cell | 1400 | - | Ref. 12 |
| 0.5 *C* | 1000 | 83.8 | Ah-level Pouch cell | 1000 | 371 | Ref. 13 |
| 0.2 *C* | 60 | 91.2 | Ah-level pouch cell | 600 | - | Ref. 14 |
| 0.2 *C* | 400 | 85.1 | Ah-level pouch cell | 540 | - | Ref. 15 |
| 1 *C* | 70 | 88 | Ah-level pouch cell | 450 | 427 | Ref. 16 |
| 3 *C* | 300 | 79.5 | Pouch cell | 748 | - | Ref. 17 |
| 0.1 *C* | 80 | 88.7 | Pouch cell | 2988 | 340.7 | Ref. 18 |
| 0.2 *C* | 120 | 89 | Pouch cell | 3380 | - | Ref. 19 |
| 1 *C* | 400 | 88 | Pouch cell | 810 | 261 | Ref. 20 |
| 0.1 *C* | 100 | 83.6 | Pouch cell | - | - | Ref. 21 |

| | | | | | | |
|---|---|---|---|---|---|---|
| 0.5 *C* | 412 | 70 | Pouch cell | 400 | - | Ref. 22 |
| 1 *C* | 160 | 80 | Pouch cell | 400 | - | Ref. 23 |
| 1 *C* | 300 | 60.5 | pouch cell | 1000 | 288.4 | Ref. 24 |
| 0.1 *C* | 100 | 58.1 | Coin cell | 2700 | - | Ref. 25 |
| 0.5 *C* | 145 | 80 | Coin cell | 3413 | - | Ref. 26 |
| 0.5 *C* | 300 | 57.1 | Coin cell | - | - | Ref. 27 |
| 0.5 *C* | 300 | 76.3 | Coin cell | 3211 | - | Ref. 28 |
| 0.1 *C* | 98 | 95.1 | Coin cell | 450 | 401.5 | Ref. 29 |
| 0.33 *C* | 300 | 86.3 | Coin cell | 2000 | - | Ref. 30 |
| 0.5 *C* | 150 | 83.7 | Coin cell | 1500 | - | Ref. 31 |
| 1 *C* | 500 | 80 | Coin cell | 2890 | - | Ref. 32 |
| 0.5 *C* | 200 | 85.2 | Coin cell | 602 | - | Ref. 33 |
| 0.5 *C* | 100 | 83.2 | Coin cell | 450 | - | Ref. 34 |
| 0.2 *C* | 100 | 77.3 | Coin cell | 1382 | - | Ref. 35 |
| 0.1 A $g^{-1}$ | 100 | 80.2 | Coin cell | 1700 | - | Ref. 36 |
| 1 *C* | 200 | 72.3 | Coin cell | 2100 | 466 | Ref. 37 |

**Supplementary References**


1. Wen, B. et al. Ultrafast ion transport at a cathode–electrolyte interface and its strong dependence on salt solvation. Nat. Energy 5, 578-586 (2020).
2. Wang, A. A., Gunnarsdottir, A. B., Fawdon, J., Pasta, M., Grey, C. P., & Monroe C. W. Potentiometric MRI of a superconcentrated lithium electrolyte: testing the irreversible thermodynamics approach. *ACS Energy Lett.* **6**, 3086-3095 (2021).
3. Hope, M. A. *et al.* Selective NMR observation of the SEI-metal interface by dynamic nuclear polarisation from lithium metal. *Nat. Commun.* **11**, 2224 (2020).
4. Yan, C. *et al.* Dual-layered film protected lithium metal anode to enable dendrite-free lithium deposition. *Adv. Mater.* **30**, 1707629 (2018).
5. Lin, L. *et al.* Li-rich $Li_2[Ni_{0.8}Co_{0.1}Mn_{0.1}]O_2$ for anode-free lithium metal batteries. *Angew. Chem. Int. Ed.* **60**, 8289-8296 (2021).
6. Chen, K. *et al.*, Material–electrolyte interfacial interaction enabling the formation of an inorganic-rich solid electrolyte interphase for fast-charging Si-based lithium-ion batteries. *Energy Environ. Sci.* **17**, 2631-2641 (2024).
7. Li, Z. *et al.*, Towards industrial applications: Ultra-stable silicon-based pouch cell conducted via a lithiated polymer binder over a wide temperature range from −25°C to 25 °C. *Nano Energy* **125**, 109619 (2024).
8. Zhuang, X. *et al.*, Interphase regulation by multifunctional additive empowering high energy lithium-ion batteries with enhanced cycle life and thermal safety. *Angew. Chem. Int. Ed.* **63**, e202315710 (2024).
9. Zhao, W. *et al.*, Modulation and quantitative study of conformal electrode-electrolyte interfacial chemistry toward high-energy-density $LiNi_{0.6}Co_{0.2}Mn_{0.2}O_2$‖SiO-C pouch cells. *Energy Storage Mater.* **53**, 424-434 (2022).
10. Sung, J. *et al.*, Subnano-sized silicon anode via crystal growth inhibition mechanism and its application in a prototype battery pack. *Nat. Energy* **6**, 1164-1175 (2021).
11. Hu, L. *et al.* Gradient H-bonding binder enables stable high-areal-capacity Si-based anodes in pouch cells. *Adv. Mater.* **33**, 2104416 (2021).
12. Fang, Q. *et al.* Interfacial degradation of silicon anodes in pouch cells. *Energy Environ. Sci.* **17**, 6368-6376 (2024).
13. Wang, H. *et al.* Unleashing the potential of high-capacity anodes through an interfacial prelithiation strategy. *ACS Nano*, **17**, 21850−21864 (2023).
14. Gao, C. *et al.* Hard–soft segment synergism binder facilitates the implementation of practical SiC600 electrodes. *Adv. Energy Mater.* **13**, 2302411 (2023).
15. Qian, Y., Liang, Y., Zhang, W., Xi, B., & Lin, N. Thermal polymerization of ion-modified carbon dots into multi-functional LiF-carbon interface for stabilizing SiO anode. *Energy Storage Mater.* **63**, 102996 (2023).
16. Liu, X. *et al.* Prelithiated Li-enriched gradient interphase toward practical high-energy NMC-silicon full cell. *ACS Energy Lett.* **6**, 320-328 (2020).
17. Lee, T. *et al.*, Suppressing deformation of silicon anodes via interfacial synthesis for fast-charging lithium-ion batteries. *Adv. Energy Mater.* **13**, 2301139 (2023).
18. Liu, T. *et al.* Recycled micro-sized silicon anode for high-voltage lithium-ion

batteries. *Nat. Sustain.* **7**, 1057–1066 (2024).
19. Li, A. *et al.* High voltage electrolytes for lithium-ion batteries with micro-sized silicon anodes. *Nat. Commun.* **15**, 1206 (2024).
20. Seo, J. *et al.* Mechanical shutdown of battery separators: Silicon anode failure. *Nat. Commun.* **15**, 10134 (2024).
21. Chen, Y. *et al.* Enabling uniform and accurate control of cycling pressure for all-solid-state batteries. *Adv. Energy Mater.* **14**, 2304327 (2024).
22. Ghaur, A. *et al.* Effective SEI formation via phosphazene-based electrolyte additives for stabilizing silicon-based lithium-ion batteries. *Adv. Energy Mater.* **13**, 2203503 (2023).
23. Weiling, M. *et al.* Mechanistic understanding of additive reductive degradation and SEI formation in high-voltage NMC811||$SiO_x$-containing cells via operando ATR-FTIR spectroscopy. *Adv. Energy Mater.* **14**, 2303568 (2024).
24. Xu, C. *et al.* Efficient implementation of kilogram-scale, high-capacity and long-life Si-C/TiO2 anodes. *Energy Storage Mater* **56**, 319-330 (2023).
25. Huo, H. *et al.* Chemo-mechanical failure mechanisms of the silicon anode in solid-state batteries. *Nat. Mater.* **23**, 543–551 (2024).
26. Pan, H. *et al.* Asolid-state lithium-ion battery with micronsized silicon anode operating free from external pressure. *Nat. Commun.* **15**, 2263 (2024).
27. Zhang, Z. *et al.* An all-electrochem-active silicon anode enabled by spontaneous Li–Si alloying for ultra-high performance solid-state batteries. *Energy Environ. Sci.* **17**, 1061-1072 (2024).
28. Cheng, Z. *et al.* Enhanced cycleability of micron-size silicon anode by in situ polymerized polymer electrolyte. *Adv. Funct. Mater.*, 2408145 (2024).
29. Yang, C. *et al.* Roll-to-roll prelithiation of lithium-ion battery anodes by transfer printing. *Nat. Energy* **8**, 703-713 (2023).
30. Zhu, T. *et al.* Formation of hierarchically ordered structures in conductive polymers to enhance the performances of lithium-ion batteries. *Nat. Energy* **8**, 129-137 (2023).
31. Tian, YF. *et al.* Tailoring chemical composition of solid electrolyte interphase by selective dissolution for long-life micron-sized silicon anode. *Nat. Commun* **14**, 7247 (2023).
32. Tan, D. *et al.* Carbon-free high-loading silicon anodes enabled by sulfide solid electrolytes. Science **373**, 1494-1499 (2021).
33. Lee, J. *et al.* Dry pre-lithiation for graphite-silicon diffusion-dependent electrode for all-solid-state battery. *Adv. Energy Mater.* **13**, 2300172 (2023).
34. Meng, Q. *et al.* A Functional prelithiation separator promises sustainable high-energy lithium-ion batteries. *Adv. Energy Mater.* **13**, 2300507 (2023).
35. Li, Z. *et al.* Engineering prelithiation of polyacrylic acid binder: A universal strategy to boost initial Coulombic efficiency for high-areal-capacity Si-based anodes. *Adv. Funct. Mater.* **32**, 2206615 (2022).
36. Li, Y. Qian, Y. Zhao, Y. Lin, N. & Qian, Y. Revealing the interface-rectifying functions of a Li-cyanonaphthalene prelithiation system for SiO electrode. *Sci. Bull.* **67**, 636-645 (2022).

37. Ma, Q. *et al.* A self-driven alloying/dealloying approach to nanostructuring micro-silicon for high-performance lithium-ion battery anodes. *Energy Storage Mater.* **34**, 768-777 (2021).
